# Structure‐Guided Extremophile Genome Mining Expands the PETase Landscape and Reveals PET‐Hydrolysing True Lipase Lineages

**DOI:** 10.1111/1751-7915.70432

**Published:** 2026-08-21

**Authors:** Rubén Muñoz‐Tafalla, José M. González‐Romero, Paula Vidal, Laura Fernandez‐Lopez, Ana Robles‐Martín, David Almendral, José L. Gonzalez‐Alfonso, Francisco J. Plou, Manuel Ferrer, Víctor Guallar, Rafael Bargiela

**Affiliations:** ^1^ PhD Program in Biotecnology, Faculty of Pharmacy and Food Sciences Universitat de Barcelona (UB) Barcelona Spain; ^2^ Barcelona Supercomputing Center (BSC) Barcelona Spain; ^3^ Instituto de Catalisis y Petroleoquimica (ICP), CSIC Madrid Spain; ^4^ Institució Catalana de Recerca i Estudis Avançats (ICREA) Barcelona Spain

**Keywords:** extremophiles, genome mining, lipases, PET hydrolases, plastic biodegradation, structural modelling

## Abstract

Poly(ethylene terephthalate) (PET) hydrolases are enzymes primarily within the polyesterase–cutinase branch of the α/β‐hydrolase superfamily, whereas the contribution of true lipases to PET hydrolysis remains poorly explored. Here, we report the genome mining results of 18,082 extremophilic microorganisms, combined with structural modelling, enzyme–substrate simulations and experimental validation, which enables the identification of PET‐active true lipases. Two true lipases, Lip_Bv_ and Lip_Sh1_, hydrolyse PET substrates and homologous enzymes within their respective clusters also retain PET‐hydrolysing activity, supporting the existence of specific lipase lineages associated with PET hydrolysis. Structural analyses suggest that differences in loop organization and active‐site accessibility may contribute to the observed PET‐hydrolysing activity and hydrolysis product profiles. These lipase lineages clustered separately from 1322 putative PETase homologues from the same extremophile genomes, while all groups remained distinct from previously characterized PETases. These findings expand the evolutionary diversity of PET‐hydrolysing enzymes in extremophiles.

## Graphical abstract

Genome mining of extremophiles revealed previously unknown PET‐hydrolising true lipases. Structural and functional analyses identified determinants of dual lipase–PETase activity and expanded the repertoire of microbial biocatalysts available for sustainable PET recycling.

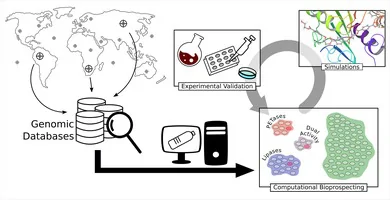

## 1 Introduction

Poly(ethylene terephthalate) (PET) is among the most widely produced synthetic polymers worldwide and is mainly used in packaging and textiles, but its chemical inertness and high crystallinity promote its long‐term accumulation in terrestrial and marine ecosystems (Galloway et al. 2017; Geyer et al. 2017; Pinaeva and Noskov 2024). Current PET recycling methods are often energy intensive and are performed under harsh conditions, limiting their sustainability (Ragaert et al. 2017; Priyadarshini et al. 2022), whereas the discovery of polyester‐hydrolysing enzymes has provided new opportunities for sustainable PET recycling and upcycling (Sui et al. 2023; Makryniotis et al. 2024; Pérez‐García et al. 2025). The identification of a PET‐hydrolysing enzyme from *Ideonella sakaiensis* marked a milestone, as it demonstrated that PET can be enzymatically hydrolysed under mild, near‐ambient conditions (Yoshida et al. 2016). Since then, the number of experimentally characterized PET‐hydrolysing proteins has steadily increased. As of 22 July 2026, 332 non‐redundant experimentally characterized PET‐hydrolysing proteins were catalogued in the PANDA (Ahituv et al. 2025), Plastics‐Active Enzymes Database (PAZy) (Buchholz et al. 2022), the Plastics Microbial Biodegradation Database (PMBD) (Gan and Zhang 2019) and PlasticDB (Gambarini et al. 2022), after redundancy removal (see Methods), while the known diversity of PET‐hydrolysing enzymes continues to expand through informatics‐based platforms for identifying microbial species with plastic‐degrading potential and artificial intelligence‐driven structural predictions (Buchholz et al. 2022; Gambarini et al. 2022; Ahituv et al. 2025; Alam et al. 2025; Hong et al. 2025). Moreover, many of these enzymes inefficiently degrade highly crystalline PET and lack the thermostability required for industrial applications, driving the search for new robust PET‐hydrolysing enzymes, including through the exploration of thermophile genomes (Gargano et al. 2025; Hu et al. 2025) and structure‐based protein language models (Wu et al. 2025) and the engineering of existing enzymes to improve catalytic properties (Wei and Zimmermann 2017a, 2017b; Zhu et al. 2022; Thomsen et al. 2023).

Regardless of their origin or performance, PETases share common structural features with other members of the α/β‐hydrolase fold family, including those within the polyesterase‐lipase‐cutinase family, in which the catalytic serine is embedded within the conserved Gly‐X₁‐Ser‐X_2_‐Gly motif, often referred to as the lipase box (Austin et al. 2018). Structural knowledge of PETases has enabled their classification into distinct families on the basis of differences in active site architecture and substrate‐binding clefts. PETases are generally divided into type I and type II enzymes, with type II enzymes further subdivided into subtypes IIa and IIb (Herrero Acero et al. 2011; Sulaiman et al. 2012; Duan et al. 2023; Wei et al. 2025). Type I PETases resemble classical cutinases, such as leaf‐branch compost cutinase (LCC) (Tournier et al. 2020), and possess a relatively narrow substrate‐binding cleft stabilized by a single disulphide bond, whereas type II PETases, such as IsPETase, contain extended loop regions and disulphide bonds that create a larger binding cleft capable of accommodating larger PET chains. These structural differences can affect substrate positioning and catalytic efficiency. In addition, outside of the PETase family, aromatic residues involved in terephthalate (TPA) recognition strongly influence PET binding and catalysis. Consequently, sequence similarity alone is insufficient to reliably identify functional PETases, as subtle structural features, including active site cleft geometry, substrate‐binding subsites and surface hydrophobicity, play critical roles in PET recognition and enzyme catalysis (Austin et al. 2018; Berselli et al. 2021; García‐Meseguer et al. 2023; Liu et al. 2023). Accordingly, these features have been extensively investigated through structural modelling and enzyme–substrate simulations, enabling the assessment of active site architecture, fold compatibility with PET‐hydrolysing activity and the mechanistic bases of substrate binding and catalysis beyond what can be inferred from sequence analysis alone (Markus et al. 2023; Stockinger et al. 2025; Tripathi et al. 2025; Xiang et al. 2025).

These analyses, including structural modelling and enzyme–substrate simulations, have also extended beyond classical PETases to the broader polyesterase‐lipase‐cutinase family. A recent study analysed more than 25,000 nonredundant α/β‐hydrolase sequences from the ESTHER database and refined a curated dataset of 2064 representatives belonging to the polyesterase‐lipase‐cutinase family that were organized into 170 clusters, representing the diversity of this enzyme family (Seo et al. 2025). From this analysis, three previously unexplored clusters (C3, C25 and C158) enriched in polyesterases with strong PET‐depolymerization potential were identified (Seo et al. 2025; Seo and Wei 2025), highlighting that polyesterases are promising, novel enzymes with PET‐hydrolysing activities. Similar conclusions have also been reached for cutinases, where multiplexed ancestral sequence reconstruction (mASR) and exploration of evolutionary sequence space elucidated ancestral variants with a broad range of PETase activities and revealed previously unexplored regions of the PETase fitness landscape (Vongsouthi et al. 2025). However, this type of large‐scale exploration has not yet been extended to lipases, one of the largest and most diverse enzyme groups within the α/β‐hydrolase superfamily. One reason why true lipases have received comparatively little attention in plastic biodegradation is that they differ fundamentally from polyesterases and cutinases (Guo et al. 2024). Whereas cutinases possess solvent‐exposed active sites that can readily attack insoluble polyester surfaces, most true lipases hydrolyse long‐chain triglycerides at lipid–water interfaces and frequently rely on interfacial activation mediated by a mobile lid domain. Consequently, true lipases have generally been regarded as less suitable candidates for PET hydrolysis, despite sharing the α/β‐hydrolase fold and catalytic machinery with polyesterases.

Lipolytic enzymes have traditionally been classified into several families on the basis of sequence similarity, with the largest group corresponding to Family I (‘true lipases’), which hydrolyse long‐chain triglycerides. For example, *Candida antarctica* lipase B (CalB), a member of lipase Family I.V, hydrolyses triacylglycerols and PET‐derived oligomers such as bis(2‐hydroxyethyl) terephthalate (BHET) and mono(2‐hydroxyethyl) terephthalate (MHET) but does not degrade PET (Nechwatal et al. 2006; Wang et al. 2008; Eberl et al. 2009; Maurya et al. 2020; Świderek et al. 2023). A lipase from *Thermomyces lanuginosus*, which is also a member of Family I.V, was reported to hydrolyse PET and the oligomeric substrate 3PET, although only in the presence of plasticizers or detergents, possibly because of interfacial activation (Eberl et al. 2009). Similarly, a lipase from *Aspergillus oryzae* was shown to hydrolyse PET short fibres but not PET films (Wang et al. 2008). In addition, lipases from *Candida cylindracea* (CcL) and *Pseudomonas* species (PsL) degraded poly(propylene terephthalate) (PPeT) nanoparticles but not PET itself (Herzog et al. 2006). More recently, Lip_MRD9_, which belongs to subfamily I.IV of the largest bacterial lipase family (Family I), was shown to hydrolyse triglycerides of different chain lengths and natural oils while also degrading both film and nanoparticle PET, as well as PET‐derived oligomers (Vidal et al. 2024). In addition, PET‐hydrolysing activity has been reported for other α/β‐hydrolases in addition to classical polyesterases and lipases. For instance, a feruloyl esterase‐like enzyme (TmFae‐PETase) identified from the gut microbiome of plastic‐degrading mealworms was shown to depolymerize PET into mono(2‐hydroxyethyl) terephthalate (MHET) and TPA, whereas canonical PETases such as IsPETase, FsC and LCC also hydrolyse ferulic acid esters, suggesting possible evolutionary links between lignocellulose‐ and PET‐hydrolysing activities (Mamtimin et al. 2024).

Taken together, these observations indicate that PET‐hydrolysing activity has emerged independently in several lipase families, raising the question of whether PET‐hydrolysing true lipases are merely sporadic functional variants or members of broader, previously unrecognized lipase lineages that have escaped systematic discovery. Resolving this question has become increasingly important as enzymatic depolymerization emerges as a promising strategy for sustainable recycling of a broad range of natural and synthetic polyesters (Prieto 2016). In this context, lipolytic enzymes are increasingly being implicated in the degradation of synthetic polyesters beyond PET, including polycaprolactone (PCL), polyurethane (PUR) and poly(butylene adipate‐co‐terephthalate) (PBAT) (Guo et al. 2024). This broader substrate scope further highlights the catalytic versatility of lipolytic α/β‐hydrolases and supports the search for previously unrecognized polymer‐degrading lipases.

In this work, rather than focusing on the characterization of individual enzymes, we investigated whether PET‐hydrolysing activity is associated with previously unrecognized evolutionary lineages of true lipases. To address this question, we developed a computational and structure‐guided genome‐mining pipeline to identify candidate true lipases with the potential to hydrolyse both lipids and PET from 18,082 extremophilic microbial genomes. Our approach combined large‐scale genome mining, structural modelling, molecular docking and enzyme–substrate simulations to prioritize candidates on the basis of active site architecture and substrate compatibility. Furthermore, the retrieved true lipases were analysed together with PETase homologues identified in the same genomes to place them within the broader evolutionary landscape of PET‐hydrolysing enzymes. Experimental characterization confirmed the PET‐hydrolysing activity of multiple true lipases belonging to previously unrecognized evolutionary lineages, highlighting extremophilic microorganisms as a promising reservoir for the discovery of additional PET‐hydrolysing enzymes.

## 2 Methods

### 2.1 Large‐Scale Mining of PET‐Hydrolysing Hydrolases in Extremophilic Genomes

Metadata from the Genome Taxonomy Database (GTDB) v226 (Parks et al. 2020, 2025) (https://gtdb.ecogenomic.org/) was downloaded from their data server (https://data.gtdb.aau.ecogenomic.org/), including information for a total of 732,477 genomes. From this dataset, genomes from cultivable extremophiles, bacteria and archaea isolated from environments classified as extreme were selected, yielding a curated set of 18,082 genomes (Table S1b). The selected genomes were subsequently downloaded using NCBI Datasets (O'Leary et al. 2024), and gene and protein annotation was performed using Prokka (Seemann 2014) when not available at the GTDB. To identify PET‐hydrolysing enzymes, two complementary reference datasets were compiled. The first included a reference dataset of 48 experimentally validated true lipases, defined as enzymes capable of hydrolysing long‐chain triglycerides, distributed across 13 lipolytic enzyme families according to the classifications of Arpigny and Jaeger (1999), Fischer (2003) and Hitch and Clavel (2019), including Families I, III, XII, XVI, XVII, XVIII, XIX, XXI, XXIII, XXV, XXVIII, XXIX and XXXII (Arpigny and Jaeger 1999; Fischer 2003; Hitch and Clavel 2019). The second dataset included 226 PETase‐related proteins retrieved from four different databases, PANDA (Ahituv et al. 2025), PAZy (Buchholz et al. 2022), PMBD (Gan and Zhang 2019) and PlasticDB (Gambarini et al. 2022), including PETase homologue sequences reported in recent large‐scale bioprospecting studies (Alam et al. 2025; Seo et al. 2025; Wu et al. 2025). The complete list of reference sequences used in this study is provided in Table S1a. These reference datasets were used as queries to search for homologous proteins in the selected extremophilic genomes. Protein similarity searches were performed against all annotated proteins using DIAMOND v2.1.10.164 (Buchfink et al. 2021), applying a threshold of ≥ 60% sequence identity and a minimum alignment length of 100 amino acids to ensure adequate sequence coverage. This threshold was selected on the basis of previous studies indicating that sequence identities greater than 40% are generally sufficient to preserve functional similarity according to Enzyme Commission (EC) numbers among homologous sequences (Addou et al. 2009; Pearson 2013). A more stringent cutoff was deliberately adopted to prioritize specificity over sensitivity during genome mining, thereby reducing the inclusion of distantly related homologues with potentially divergent substrate specificities. Although this approach may exclude more remote candidates with novel catalytic activities, it provides greater confidence in the functional assignment of candidate PET‐hydrolysing true lipases. Hence, conservative filters were established to minimize false‐positive functional assignments (Rost 1999; Jia et al. 2023). The resulting candidate sequences were further processed using in‐house Perl scripts and filtered for the presence of signal peptides using SignalP v5.0 (Almagro Armenteros et al. 2019), as true lipases are frequently secreted enzymes or associated with the cell envelope and therefore often contain N‐terminal signal peptides (Jaeger 1998; Arpigny and Jaeger 1999).

To determine whether the new sequences identified by the homology search differed from those of other potential previously discovered PETases, a sequence similarity network (SSN) analysis was carried out with the sequence sets used in similar studies (Seo et al. 2025). Within selected clusters, particularly those containing Lip_Sh1_, representative PETase candidates were prioritized for further experimental validation. For this purpose, the three‐dimensional structures of candidates belonging to the cluster were predicted using Boltz 2.1.1 (Passaro et al. 2025), as it better predicted the folding of the IDR regions of the protein than AlphaFold 2.0 (Jumper et al. 2021) (model 1.1.1). Owing to low confidence in the C‐terminal region, only the catalytic α/β‐hydrolase fold domain, identified using InterProScan v5.69 (Jones et al. 2014), was considered. The predicted structures were subsequently subjected to energy minimization using Rosetta FastRelax with five repeats and the ref2015 scoring function (Leman et al. 2020).

### 2.2 Structure‐Based Screening and Enzyme–Substrate Simulations

Structural models of selected sequences were generated using AlphaFold 2.0 (Jumper et al. 2021) (model 1.1.1), and residues located at the sequence termini with predicted local distance difference test (pLDDT) scores of less than 90 were removed. Protein structures were subsequently prepared using Schrödinger's Protein Preparation Wizard (Madhavi Sastry et al. 2013), where hydrogen bonding networks and protonation states were optimized with PropKa (Olsson et al. 2011) at pH 7.0 and disulphide bonds were introduced when needed. The resulting models representing different folding scaffolds were aligned with experimentally determined structures to identify potential ion binding sites. To evaluate the potential of the selected hydrolases to recognize triglycerides, which is indicative of lipase activity, and PET oligomers, which is indicative of PET‐hydrolysing activity, enzyme–substrate interactions were investigated using structural modelling and enzyme–substrate simulations. Two model substrates were used: glyceryl tridecanoate (Tri‐C10) and ETETETETE (where E = ethylene and T = terephthalate according to the nomenclature of Schubert et al. 2022). Substrate parameter files were prepared using Jaguar software (Cao et al. 2024) to obtain appropriate quantum mechanics‐derived atomic charges. Following preparation, the substrates were docked into the active site of the enzymes using GLIDE (Friesner et al. 2006), with ExtraPrecision (XP) accuracy. The docking grid box in each case was centred on the alcoholic oxygen of the catalytic serine. The selected starting pose, among the top 100 docking poses, was the pose showing the shortest catalytic distance between the reactive oxygen of the serine and any of the ester bond‐forming carbon atoms of the substrate. Protein energy landscape exploration (PELE) software (Borrelli et al. 2005) was used to examine the interactions between the enzymes and the substrates. PELE is a heuristic Monte Carlo simulation framework specifically designed to explore protein–substrate‐induced fit interactions and has been widely applied in drug design and enzyme engineering. Starting from the best GLIDE pose, a PELE simulation was performed for each system using 112 computing cores, each running 10 epochs of 100 Monte Carlo steps. The simulation results were analysed using a Boltzmann distribution‐based metric, referred to in this work as the catalytic binding free energy (CBFE). The CBFE was obtained by integrating the Boltzmann probabilities of each accepted step, which depend on the total energy of the system, weighted by the interaction energy and considering only poses classified as catalytic (Equation 1).

(1) $$P_{i}=\frac{e^{\frac{-E_{i}}{\mathrm{KT}}}}{Q};Q=\sum_{i}^{N}e^{\frac{-E_{i}}{\mathrm{KT}}};\left\langle E^{b}\right\rangle =\sum_{i}^{N}P_{i}\cdot E_{i}^{b}$$

Equation (1) The total energy and KT value are used to calculate the probability of the system reaching the i‐th pose (P_i_), where *Q* is the partition function for a simulation consisting of N poses (in this case, only catalytic poses are considered for the calculation). E^b^ represents the binding free energy, which is calculated by weighting the probability of each pose by its interaction energy obtained from PELE.

Catalytic poses were defined on the basis of distance criteria between protein–substrate reactive atoms and hydrogen bonds between residues of the catalytic triad. The computational parameters and thresholds applied for docking, the PELE simulations and candidate selection are summarized in Table 1. Specifically, the protein–substrate catalytic distance was defined as the shortest distance between the oxygen atom of the catalytic serine and any suitable ester bond of the substrate. Distances within the catalytic triad included the serine oxygen and the unprotonated nitrogen of histidine, as well as the interaction between the protonated nitrogen of histidine and the closer oxygen atoms of the aspartic acid residue.

**TABLE 1:** Classification of lipase families in the final candidate set.

Family	Number of candidates	Length (AA)	LID domain	Disulphide bond(s)	Ion binding site
I.I	22	280–290	Yes	Yes	No
I.IV	8	~180	No	No	No
I.VI	11	270–350	Yes	No	Calcium and Zinc
I.VIII	1	430	Yes	Yes	No
XXVIII	8	~420	No	Yes	No

### 2.3 Gene Synthesis, Heterologous Expression and Purification of Selected Enzymes

The gene sequences encoding the target enzymes (see accession numbers and sequences in Table S1e) were used as templates for gene synthesis. The sequences were synthesized by GenScript Biotech (GenScript Biotech, EG Rijswijk, The Netherlands) and codon optimized to maximize expression in *Escherichia coli* . In brief, before gene synthesis, the sequence was analysed for the presence of a signal peptide using the SignalP‐5.0 tool (Almagro Armenteros et al. 2019). The genes, excluding the signal peptide when present, were flanked by *BamHI* and *HindIII* (stop codon) restriction sites and inserted into a pET‐45b(+) expression vector with an ampicillin selection marker (GenScript Biotech, Rijswijk, The Netherlands). This plasmid, which was introduced into *E. coli* ArcticExpress (DE3), supports the expression of N‐terminal His_6_‐fusion proteins, with the final amino acid sequence of the synthetic protein being MAHHHHHHVGTGSNDDDDKSPDPM‐X (where X corresponds to the original sequence of the target enzyme without the signal peptide). After synthesis, soluble N‐terminal His_6_‐tagged proteins were produced in *E. coli* and purified at 4°C by Ni affinity chromatography, ultrafiltration, dialysis and size‐exclusion FPLC, yielding final protein preparations concentrated to 10 mg mL^−1^ with > 98% purity as determined by SDS‐polyacrylamide gel electrophoresis (SDS–PAGE) on 12% gels using a Mini PROTEAN electrophoresis system (Bio‐Rad, Madrid, Spain) (Laemmli 1970), as previously described (Vidal et al. 2024).

### 2.4 Lipase Activity Assay With Triglycerides, Natural Oils and Fatty Acid Esters

The activities of the enzymes with lipidic substrates, including triglycerides, natural oils and fatty acid esters, were determined through the quantification of released non‐esterified fatty acids (NEFAs), as previously described (Vidal et al. 2024). In brief, reactions were performed in 2‐mL safe‐lock Eppendorf polypropylene tubes (ref. 0030 120.094; Greiner Bio‐One GmbH, Kremsmünster, Austria) containing 40 mM 4‐(2‐hydroxyethyl)1‐1piperazineethanesulfonic acid (HEPES)–3 mM CaCl_2_ buffer (pH 7.0), 24 mM substrate and 0.14 mg mL^−1^ enzyme and incubated at 40°C for 24 h in a thermoshaker (Thermomixer Comfort, Eppendorf AG, Hamburg, Germany) at 950 rpm. Hydrolysis was quantified by measuring the release of NEFAs using a NEFA Kit (FUJIFILM Wako Chemicals Europe GmbH, Neuss, Germany) according to the manufacturer's instructions. The absorbance was measured at 550 nm using a Synergy HT Multi‐Mode Microplate Reader (BioTek Instruments, Winooski, VT, USA) and Gen5 2.00 software. Enzyme activity was calculated using a calibration curve prepared with a NEFA standard (oleic acid, ref. 29,124–2; Merck Life Science S.L.U., Madrid, Spain). All experiments were performed in triplicate and included controls without enzyme.

### 2.5 PET Hydrolysis Assays Using Nanosized and Powdered PET

In vitro PET hydrolysis assays using nanosized PET (nPET) and PET powder (pPET) were performed as previously described (Robles‐Martín et al. 2023). Briefly, standardized nPET substrates were prepared from amorphous PET (Goodfellow Cambridge, Huntingdon, UK; ref. ES301445), while PET powder (Goodfellow Cambridge, Huntingdon, UK; ref. ES30PD000132) was obtained from the same supplier. The physicochemical properties of nPET (diameter 1–5.5 nm; crystallinity 1.3%; ΔHc = 32.8 J g^−1^; Tg = 73.7°C; Tm = 246.7°C; and Tc = 200.6°C) have been reported previously (Robles‐Martín et al. 2023). PET powder (pPET) consisted of particles < 300 μm with > 40% crystallinity. Reactions (50 μL) were carried out in 20–40 mM HEPES buffer containing 1.5–3 mM CaCl_2_ (pH 7.0) with enzyme and substrate concentrations of 0.5–1 mg mL^−1^ and 1.55–1.65 mg mL^−1^ (nPET) or 7 mg mL^−1^ (pPET), respectively, incubated at 40°C or 60°C with shaking at 950 rpm for 2–6 h. Reactions were performed in 2‐mL safe‐lock Eppendorf polypropylene tubes (ref. 0030120094) and terminated by the addition of 450 μL of dimethyl sulfoxide (DMSO) prior to high‐performance liquid chromatography (HPLC) analysis of the degradation products as previously described (Robles‐Martín et al. 2023). Degradation products were quantified by HPLC, and PET‐derived intermediates were identified by mass spectrometry, as previously described (Robles‐Martín et al. 2023; Vidal et al. 2024, 2025; Giménez‐Dejoz et al. 2026). All experiments and analyses were performed in biological triplicates and included controls without enzyme.

pPET hydrolysis assays were also performed with the benchmark PET hydrolases LCC‐variants (Tournier et al. 2020) and HotPETase (Bell et al. 2022) using purified enzymes. Protein production and purification were carried out as previously described (Vidal et al. 2025), and PET hydrolysis assays were performed under the conditions described above.

### 2.6 Enzymatic Activity Temperature and pH Dependence

The optimal temperature was determined using the lipase activity assay described above, with glyceryl trioctanoate (Tri‐C8; 24 mM) as the substrate. Reactions were performed in 2‐mL safe‐lock Eppendorf polypropylene tubes (ref. 0030 120.094; Greiner Bio‐One GmbH, Kremsmünster, Austria) in a thermoshaker (Thermomixer Comfort, Eppendorf AG, Hamburg, Germany) at 950 rpm. The reaction mixtures (50 μL) contained 40 mM HEPES–3 mM CaCl_2_ buffer (pH 7.0) and enzyme concentrations ranging from 0.05 to 0.14 mg mL^−1^, depending on the enzyme. Prior to initiating the reaction, the buffer was preequilibrated for 10 min at the indicated temperature. Reactions were carried out at temperatures ranging from 10°C to 70°C for 10 min. Hydrolysis was quantified by measuring the release of NEFAs as described above. All experiments were performed in triplicate (*n* = 3), including control reactions without enzyme to account for background signals. The optimal pH was determined using *p*‐nitrophenyl butyrate (*p*NPB) as a substrate under the following conditions: an enzyme concentration of 0.005 mg mL^−1^, a substrate concentration of 1.6 mM, a reaction volume of 100 μL of 50 mM Britton–Robinson buffer (pH 3.0–11.0), a temperature of 40°C and a reaction time of 10 min. The reactions were terminated by the addition of 50 μL of 6% Na_2_CO_3_. The absorbance at 405 nm was measured using a Synergy HT Multi‐Mode Microplate Reader (BioTek Instruments, Winooski, VT, USA). All experiments were performed in triplicate and included controls without enzyme.

### 2.7 Phylogenetic Analysis

To investigate the evolutionary relationships among PET‐hydrolysing α/β‐hydrolases, a phylogenetic analysis was performed using experimentally validated PETases, representative canonical PETases (included within the cutinase, lipase and polyesterase families), validated true lipases lacking PET‐hydrolysing activity, predicted PETase homologues identified in this study, and the PET‐hydrolysing true lipases characterized here. Protein sequences were aligned using MAFFT v7.525 (Katoh and Standley 2013). The resulting multiple sequence alignment was trimmed with trimAl v1.4.1 using the ‐gt 0.7 option (Capella‐Gutiérrez et al. 2009). Maximum‐likelihood phylogenetic reconstruction was carried out with IQ‐TREE v3.1.3 (Wong et al. 2026) using 1000 bootstrap replicates. The tree was visualized and annotated using iTOL v6 (Letunic and Bork 2024).

## 3 Results

### 3.1 Genome Mining and Simulation‐Guided Prioritization of Extremophile‐Derived PET‐Hydrolysing Lipases

To identify true lipases with potential PET‐hydrolysing activity, a reference dataset of 48 experimentally validated true lipases distributed across 13 lipolytic enzyme families was subjected to similarity searches (see Methods and Table S1a). We focused on true lipases for several reasons. First, although PET hydrolysis has been studied mainly in polyesterases and cutinases, several true lipases have been reported to hydrolyse PET, PET‐derived oligomers or structurally related synthetic polyesters. These include *C. antarctica* lipase B (CalB), lipases from *T. lanuginosus* and *Aspergillus oryzae*, *C. cylindracea* lipase (CcL), *Pseudomonas* spp. lipases (PsLs) and Lip_MRD9_ (Herzog et al. 2006; Wang et al. 2008; Eberl et al. 2009; Carniel et al. 2017; Vidal et al. 2024). Second, although cutinases and polyesterases have already been extensively bioprospected for PET degradation (Seo et al. 2025; Vongsouthi et al. 2025), bacterial true lipases remain relatively underexplored.

Esterases have not been consistently reported to hydrolyse PET and generally display a preference towards soluble short‐chain esters, whereas lipases are structurally adapted to interact with hydrophobic and water‐insoluble substrates through interfacial activation mechanisms and exposed hydrophobic regions surrounding the catalytic site (Arpigny and Jaeger 1999). This is why, as an additional filtering criterion during the search protocol, only proteins containing N‐terminal signal peptides were retained, as secretion is a common feature of extracellular bacterial lipases involved in the degradation of hydrophobic substrates (Jaeger 1998; Arpigny and Jaeger 1999). We conducted a large‐scale bioprospecting analysis of extremophilic microbial genomes from the GTDB (Parks et al. 2020, 2025). Because extremophiles have evolved under strong selective pressures (Dance 2020; Shu and Huang 2021), they represent a promising source of structurally and functionally diverse PET hydrolases that may not be detected in conventional microbial datasets. Protein sequences encoded in 18,082 extremophile genomes retrieved from the GTDB (Table S1b) were screened against a curated reference dataset of experimentally validated true lipases using sequence similarity‐based criteria (see Methods). Moreover, as stated, only proteins containing N‐terminal signal peptides were retained. This pipeline predicted 178 true lipases (Table S1c), which were subsequently dereplicated on the basis of pairwise sequence similarity, resulting in a final set of 50 nonredundant candidate sequences encoding putative true lipases (Table S1d).

The identification of only 178 candidate true lipases across 18,082 extremophile genomes likely reflects the conservative design of the screening pipeline rather than a genuine scarcity of these enzymes. First, similarity searches were performed using a highly curated reference dataset comprising only 48 experimentally validated true lipases distributed across 13 lipolytic enzyme families according to the classifications of Arpigny and Jaeger (1999), Fischer (2003) and Hitch and Clavel (2019), including Families I, III, XII, XVI, XVII, XVIII, XIX, XXI, XXIII, XXV, XXVIII, XXIX and XXXII. As demonstrated in other sequence‐based metagenomic approaches, the number of candidates retrieved is vastly dependent on the completeness and accuracy of the reference databases (Lu, Diaz, et al. 2022; Lu, Schneider, and Daniel 2022). Second, bacterial true lipases are less abundant than polyesterases, which dominate most genomic and metagenomic datasets (Arpigny and Jaeger 1999). Additionally, only proteins containing N‐terminal signal peptides were retained to identify secreted enzymes, since true lipases are typically extracellular biocatalysts that have adapted to act on insoluble hydrophobic substrates, including polyesters. This criterion inevitably reduced the explored sequence space and may have excluded intracellular PET‐hydrolysing enzymes lacking secretion signals. However, the primary objective of this study was to identify extracellular true lipases capable of hydrolysing both insoluble lipid substrates and PET, for which the presence of a signal peptide is a biologically relevant feature. Therefore, prioritizing proteins with predicted secretion signals was considered appropriate for the scope of this work. Future studies extending the search to intracellular α/β‐hydrolases may uncover additional PET‐hydrolysing true lipases with distinct biological functions or physiological roles.

The candidates displayed substantial sequence and taxonomic diversity, with a mean sequence identity of 73.7% (interquartile range [IQR] = 11.9%) relative to public database entries and the presence of both distant (percentage identity [PID] < 75%) and closely related clusters. The enzymes were distributed across three major bacterial phyla, namely, *Bacillota*, *Pseudomonadota* and *Actinomycetota*, and were affiliated with diverse genera, the most representative being *Staphylococcus*, *Bacillus*, *Halopseudomonas*, *Stutzerimonas*, *Rhodococcus*, *Pseudoalteromonas*, *Vibrio*, *Marinobacter* and *Alcanivorax* (Table S1d). These results indicate broad phylogenetic and ecological diversity, suggesting substantial structural diversity among extremophile‐derived true lipases.

**FIGURE 1:**
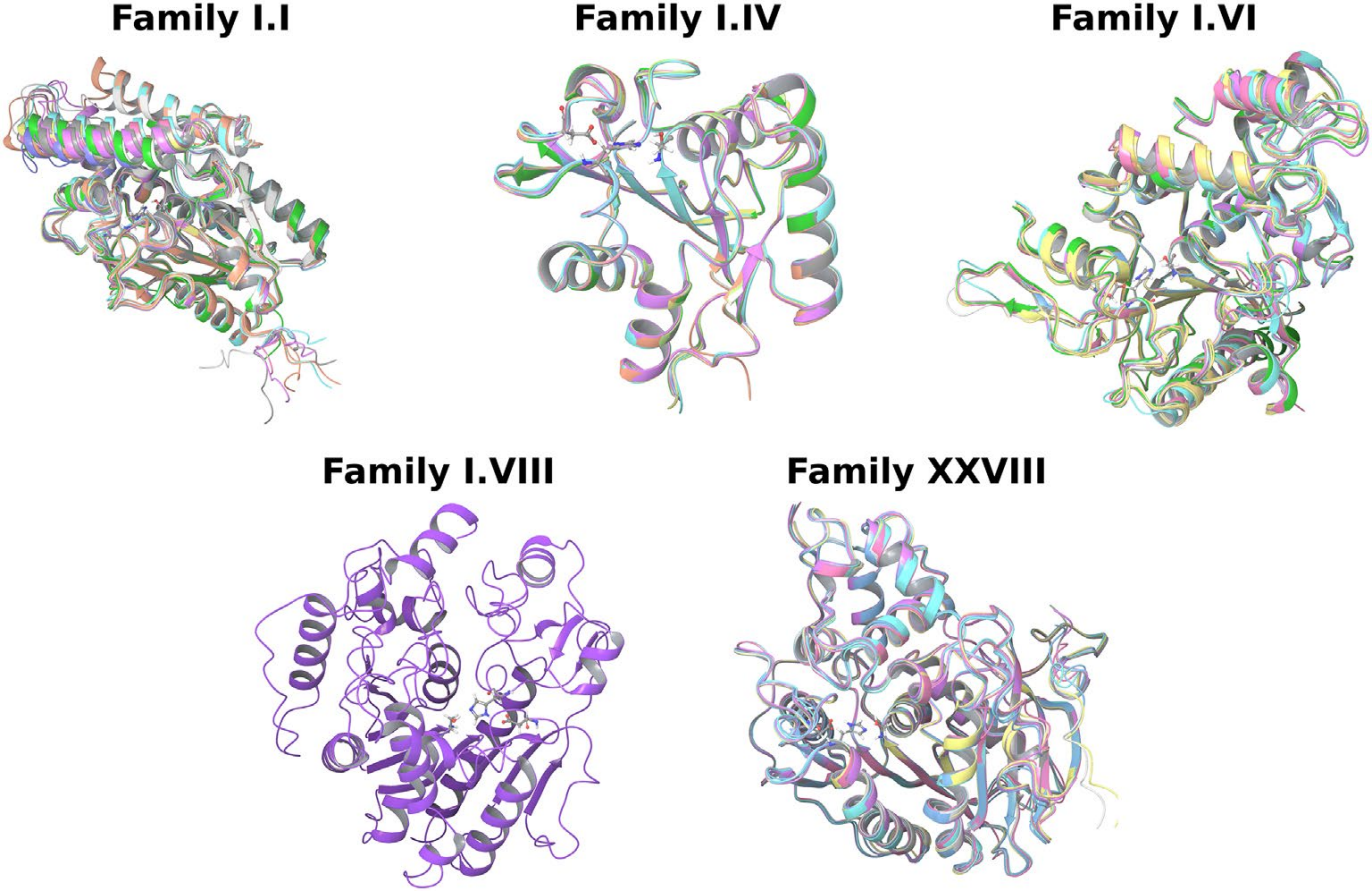
Cartoon representations of the structures of different groups of candidate lipases, with the catalytic triad shown in ball and stick representation. (Family I.I) Lipases containing a canonical LID domain. (Family I.IV) Small lipases lacking a LID domain. (Family I.VI) Lipases with an extended N‐terminal region. These candidates have sequence lengths of 700–800 amino acids; however, AlphaFold2 did not confidently model the first ~300 amino acids, which appear largely unstructured. Therefore, only the folded catalytic domain was considered for subsequent simulations. (Family I.VIII) Large lipases with a LID domain. (Family XXVIII) Large lipases lacking a LID domain.

All the candidates were subsequently subjected to structural modelling and enzyme–substrate simulations to evaluate their ability to accommodate lipid and PET‐like substrates; thus, the three‐dimensional structures of the selected presumptive true lipase candidates were predicted using AlphaFold2. Structural analysis of the 50 selected putative lipases revealed distinct folding scaffolds characteristic of five lipase families (Table 1, Figure 1, Table S1d). Although all the candidates shared the conserved α/β‐hydrolase core typical of lipolytic enzymes, they differed markedly in amino acid (AA) size (from 150 to 700), domain architecture and structural features such as lid domains, number of disulphide bonds and ion‐binding sites. Predicted disulphide bonds were detected mainly in members of Families I.I, I.VIII and XXVIII (Table 1, Table S1d).

After structural preparation and protonation of the models, including the assignment of disulphide bonds and ion‐binding sites (Table 1), docking simulations were performed using two representative substrates: Tri‐C10 and a PET tetramer with ethylene groups at both termini to preserve symmetry (ETETETETE). The PET tetramer was selected because it is relevant to PET degradation processes and has been widely used as a model substrate in computational studies aimed at characterizing PET‐hydrolysing active sites and enzyme–substrate interactions (Vidal et al. 2024). Related PET‐derived oligomers of shorter lengths, such as ETETETE, ETETE and ETE, have also been successfully employed in similar studies (Vidal et al. 2025; Giménez‐Dejoz et al. 2026). Tri‐C10 was included as a model triglyceride substrate that has typically been linked to the activity of true lipases, with the aim of identifying lipases with the capacity to hydrolyse not only natural triglycerides but also polyester‐like substrates such as PET. This stringent dual‐substrate criterion was intentionally applied to narrow the dataset to a small group of high‐confidence and functionally promising candidates for subsequent experimental validation. For each enzyme–substrate pair, the best docking pose fulfilling the catalytic distance criterion was selected as the starting point for subsequent simulations. This pose corresponded to the shortest distance between the oxygen atom of the catalytic serine and any suitable ester bond of the substrates (nonterminal ester bonds in the case of the PET tetramer and the ester bond linking the glycerol backbone to the fatty acid chains in the case of the triglyceride). Examples of the catalytic docking poses for both substrates are shown in Figure S1a,b. Following docking, only 23 of the 50 candidate enzymes displayed catalytic conformations compatible with nucleophilic attack by the catalytic serine for both substrates on the basis of the geometric and energetic criteria summarized in Table 2.

**TABLE 2:** Docking and PELE simulation parameters used for candidate prioritization.

Simulation parameter	Applied threshold/Criterion
pLDDT score threshold	> 90%
Docking distance threshold	5.0 Å
GLIDE score criteria	Lowest‐energy pose meeting distance criteria
Distance constraints	Ser–Lig = 4.5 Å; Ser‐His = 4.5 Å; His‐Asp = 3.5 Å
CBFE threshold for Tri‐C10	−15.0 kcal mol^−1^
CBFE threshold for PET tetramer	−12.0 kcal mol^−1^

As the identified candidates were expected to function as lipases, these 23 enzymes were first subjected to PELE simulations to examine the interactions between the enzymes and Tri‐C10 as a model lipid substrate. PELE software (Borrelli et al. 2005) was employed to perform a thorough quantitative analysis of catalytically competent conformations, allowing further ranking of the candidates. Figure S2a shows the interaction energy profiles for each enzyme, whereas Figure S2b summarizes the corresponding catalytic binding free energy (CBFE) rankings, which provide a comparative energetic metric to assess enzyme–substrate interactions for a given substrate.

On the basis of these results, the 15 candidates with the most favourable CBFE values were selected for subsequent simulations with the PET tetramer substrate. To assess the ability of our simulation protocol to capture catalytically competent interactions with PET substrates, we first performed PELE simulations using three well‐characterized PET hydrolases: FAST‐PETase, *Humicola insolens* cutinase (HiC) and LCC‐ICCG. FAST‐PETase is an enzyme that was engineered on the basis of a machine learning approach that has been reported to depolymerize untreated, diverse postconsumer PET materials with exceptional efficiency and high activity (Lu, Diaz, et al. 2022; Lu, Schneider, and Daniel 2022). HiC is a widely used cutinase with demonstrated PET‐hydrolysis activity that has frequently served as a high‐performing industrial benchmark for cutinase‐type PET degradation (Eugenio et al. 2021). LCC‐ICCG (an engineered variant of LCC) is a thermostabilized, high‐activity LCC derivative widely reported as a state‐of‐the‐art PET hydrolase that is commonly used as an industrially relevant benchmark, combining speed, efficiency, and scalability in enzymatic PET recycling and recently characterized by residue‐specific NMR analyses (Grga et al. 2025). The simulation results for these three enzymes with the PET tetramer substrate are shown in Figure S3a. All three enzymes display clear interaction energy minima when the substrate ester bond approaches the catalytic serine in the active site, while the catalytic triad remains in a catalytically competent conformation. These results support the ability of our computational protocol to identify productive binding modes with PET‐hydrolysing enzymes.

PELE results obtained for the 15 candidates (showing the most favourable CBFE values with TriC‐10) with the PET tetramer substrate (ETETETETE) are shown in Figure S3b. The final ranking of candidates based on the CBFE metric is shown in Figure S3c. This metric integrates the energetic and geometric parameters described above to estimate the effective binding free energy for each enzyme–substrate system. Seven candidate enzymes (MCQ4299104, TRM03865, HDZ55628, WP_000842036.1, WP_010331400.1, RGP46094 and WP_032375680.1) were predicted by PELE to productively bind to both the triglyceride substrate and the PET tetramer. Candidates with a calculated CBFE superior to that of the bibliographically characterized PETase with the lowest binding score (FAST‐PETase) were selected, ensuring that only the most promising enzymes were considered for further analysis. Two additional enzymes were included as negative controls for PET hydrolysis to validate the robustness of the computational workflow, namely, RSZ23112 and MBC29882 (Lu, Diaz, et al. 2022; Lu, Schneider, and Daniel 2022) (Family I.VI), which were predicted to bind strongly in a catalytically active pose with the triglyceride substrate but showed no productive binding in the simulations with PET.

### 3.2 Experimental Validation of Simulation‐Prioritized PET‐Active Lipases

Of the nine selected enzymes, three (MCQ4299104 and HDZ55628 from Family I.I and WP_032375680.1 from Family XXVIII) could not be expressed in soluble form and were therefore excluded from further analyses. Consequently, the PET‐hydrolysing potential of these candidates and their corresponding structural families remains unresolved pending successful heterologous expression. The remaining six enzymes were successfully expressed in soluble form and subjected to further characterization (Figure S4). Hereafter, enzyme names are abbreviated using the prefix ‘Lip’ (for lipase), followed by the initials of the genus and species of the source microorganism in subscript. Thus, these enzymes are referred to as Lip_Se_ (MBC29882), Lip_Bv_ (WP_010331400.1), Lip_Sha_ (RSZ23112), Lip_Sh1_ (TRM03865), Lip_Sa_ (WP_000842036.1) and Lip_Re_ (RGP46094). The predicted molecular masses of the N‐terminal His‐tagged recombinant proteins were 74.7 kDa (Lip_Se_‐derived construct), 22.1 kDa (Lip_Bv_‐derived construct), 81.5 kDa (Lip_Sha_‐derived construct), 83.4 kDa (Lip_Sh1_‐derived construct), 75.7 kDa (Lip_Sa_‐derived construct) and 46.6 kDa (Lip_Re_‐derived construct). The selected enzymes originated from diverse environments and belonged to the phylum Bacillota and mainly the genus *Staphylococcus*, whereas others were affiliated with *Bacillus* and *Rhodococcus* (Table S1e). The enzymes were assigned to Families I.IV, I.VI and XXVIII, with sequence identities ranging from 62.1% to 98.7% relative to their closest homologue in the reference set. Structural analysis revealed distinct folding architectures among the candidates. The four Family I.VI enzymes (Lip_Se_, Lip_Sha_, Lip_Sh1_ and Lip_Sa_) exhibited a large α/β‐hydrolase fold preceded by an extended disordered N‐terminal region, a feature consistent with that of secreted staphylococcal lipases. In contrast, Lip_Bv_ displayed a small Lip_MRD9_‐like fold (Vidal et al. 2024), characteristic of smaller bacterial lipases, whereas Lip_Re_, assigned to Family XXVIII, is a larger lipase lacking a canonical LID domain and predicted to contain a disulphide bond.

The selected recombinant enzymes were subsequently purified and evaluated for lipolytic and PET‐hydrolysing activities. For the initial functional characterization, lipase activity was evaluated using a panel of triglycerides, other ester substrates and complex natural lipids by quantifying the release of NEFAs (Table 3). All the enzymes displayed clear lipolytic activity, supporting their classification as true lipases. Lip_Bv_ showed the highest overall activity, reaching 3715 ± 9 μmol NEFA h^−1^ g^−1^ with Tri‐C8, whereas Lip_Se_ and Lip_Sh1_ also displayed high activity exceeding 3200 and 2100 μmol h^−1^ g^−1^, respectively, with medium‐chain triglycerides. In contrast, Lip_Sha_ showed moderate activity, and Lip_Re_ had the lowest overall hydrolytic activity. Most of these enzymes preferentially hydrolysed medium‐chain triglycerides and complex lipid mixtures rather than long‐chain esters and lipids, which is in agreement with the PELE predictions indicating their favourable binding to triglyceride substrates.

**TABLE 3:** Activities (μmol h^−1^ g^−1^) of enzymes with different lipid substrates.

Substrate^a^	Activity (μmol h^−1^ g^−1^)					
	Lip_Bv_	Lip_Se_	Lip_Sa_	Lip_Sh1_	Lip_Sha_	Lip_Re_
Lipase family	I.IV	I.VI	I.VI	I.VI	I.VI	XXVIII
Tri‐C8	3715 ± 9	3292 ± 13	3228 ± 70	2185 ± 22	1688 ± 6	299 ± 1
Tri‐C10	1499 ± 4	1489 ± 6	780 ± 3	1180 ± 9	561 ± 3	161 ± 1
Tri‐C14	964 ± 7	166 ± 1	116 ± 32	34 ± 1	73 ± 1	0
Coconut oil	1051 ± 1	1085 ± 7	882 ± 8	1216 ± 12	402 ± 4	68 ± 1
Palm oil	437 ± 4	241 ± 4	97 ± 2	132 ± 1	87 ± 1	13 ± 1
Olive oil	101 ± 1	110 ± 1	125 ± 10	106 ± 1	23 ± 1	6 ± 1
Ethyl myristate	151 ± 1	167 ± 1	114 ± 1	237 ± 1	82 ± 1	52 ± 1
Oleyl oleate	5 ± 1	1 ± 1	3 ± 1	8 ± 1	0	6 ± 1

^a^ Experimental conditions: [Substrate], 24 mM from stock solutions prepared in DMSO, except for ethyl myristate and oleyl oleate, which were prepared in chloroform; [Enzyme], 0.14 mg mL^−1^; buffer, 40 mM HEPES containing 3 mM CaCl_2_, pH 7.0; temperature, 40°C; shaking, 950 rpm; reaction time, 24 h. Abbreviations: Glyceryl trioctanoate (Tri‐C8), glyceryl tridecanoate (Tri‐C10) and glyceryl trimyristate (Tri‐C14).

**FIGURE 2:**
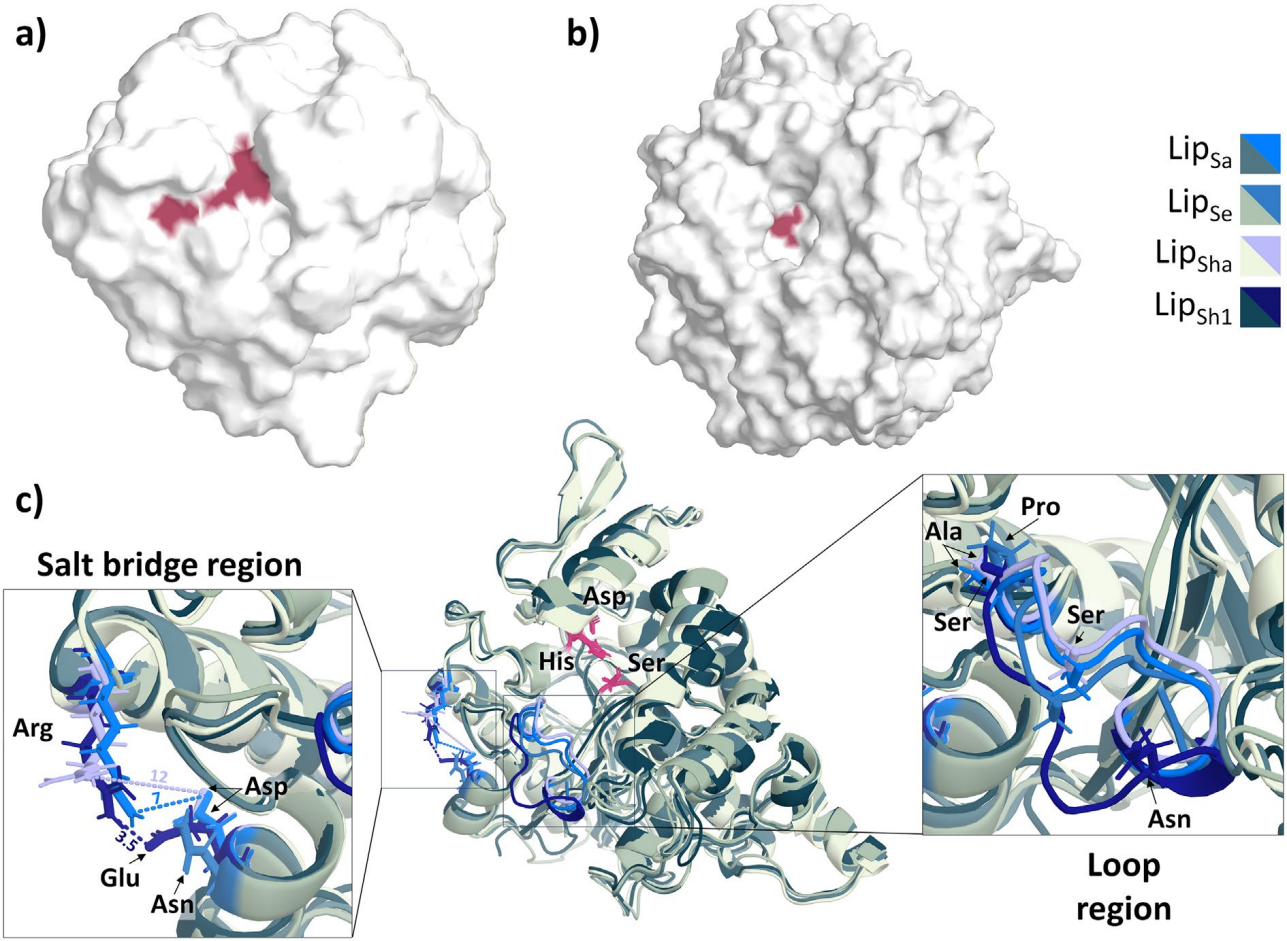
Structural features in selected candidate PET‐hydrolysing lipases. (a) Three‐dimensional structure of Lip_Bv_, with the residues of the catalytic triad highlighted in raspberry. (b) Three‐dimensional structure of Lip_Re_, with the residues of the catalytic triad highlighted in raspberry. (c) Structural comparison of members of lipase Family I.VI, with the residues of the catalytic triad highlighted in raspberry and relevant structural motifs in different colours (legend). Insets show differences in amino acids and bonds involved in a putative salt bridge (left) and a loop region located near the substrate binding pocket (right).

Structural inspection revealed notable differences in active‐site architecture among the enzymes (Figure 2). Lip_Bv_ displayed a highly exposed catalytic serine and an open active site (reddish area, Figure 2a), whereas Lip_Re_ presented a narrower and more buried catalytic pocket (Figure 2b). Within Family I.VI, Lip_Sh1_ differed from Lip_Se_, Lip_Sa_ and Lip_Sha_ by three substitutions located near the catalytic pocket (Figure 2c, middle). Two of these substitutions corresponded to residues positioned close to the catalytic serine, namely, asparagine 372 and serine 375 in Lip_Sh1_, whereas the corresponding positions in the other Family I.VI enzymes were occupied by serine, alanine or proline residues (Figure 2c, right). A third substitution involved glutamate 388 in Lip_Sh1_, replacing an aspartate residue in the other enzymes (Figure 2c, left). Structural models indicated that this glutamate is positioned near a conserved arginine residue (Arg706), forming a putative salt bridge interaction adjacent to the loop region that leads to the catalytic pocket entrance. In contrast, the corresponding residues in Lip_Sa_ and Lip_Sha_ are aspartates, whose shorter side chains are likely to hinder the formation of this interaction (Figure 2c, left).

Comparative structural models were complemented with quantitative analyses of the active‐site architecture. FPocket analysis revealed clear differences in pocket volume and hydrophobicity between Lip_Bv_ and Lip_Sh1_, as representatives of lipase families I.IV and I.VI, respectively, while solvent‐accessible surface area calculations showed that active‐site residues in Lip_Bv_ are, on average, more solvent‐exposed than those of Lip_Sh1_, consistent with the latter possessing a more buried active site. These quantitative descriptors provide additional support for the structural observations described above and are summarized in Figure S5.

Following lipolytic characterization, the selected enzymes were evaluated for PET hydrolysis at 40°C and 60°C using nanosized PET particles (nPET) and microsized PET powder (pPET), which differ in terms of substrate accessibility and crystallinity (Robles‐Martín et al. 2023). Among the soluble enzymes tested, only Lip_Bv_ and Lip_Sh1_ showed detectable PET‐hydrolysing activity (Table 4). Lip_Bv_ displayed the highest activity, reaching 2706 ± 12 μmol h^−1^ g^−1^ with nPET at 60°C and was active against both nPET and pPET at both temperatures tested. In contrast, Lip_Sh1_ showed lower activity, with a maximum of 431 ± 3 μmol h^−1^ g^−1^ with nPET at 40°C and no detectable activity at 60°C, most likely because of reduced stability or the loss of productive substrate binding at higher temperatures. Notably, Lip_Sh1_ was the only active enzyme among the Family I.VI lipases, which is consistent with structural analyses indicating the more accessible catalytic pocket in this variant (Figure 2a,b). The remaining enzymes showed no detectable PET hydrolysis under the tested conditions. PELE simulations correctly predicted the absence of productive PET binding for Lip_Se_ and Lip_Sha_, whereas the lack of activity of Lip_Sa_ and Lip_Re_ despite favourable simulation results likely reflects factors not captured by the computational workflow, such as catalytic activation barriers or enzyme stability. Overall, the agreement between the simulation and experimental data supports the predictive ability of the PELE‐based screening strategy. As a reference, the previously characterized PET‐active lipase Lip_MRD9_ (Vidal et al. 2024) was included in the assays. Although Lip_MRD9_ showed similar or higher activity towards nPET at 40°C, Lip_Bv_ displayed the highest activity at 60°C, further supporting its robust PET‐hydrolysing capability.

**TABLE 4:** Activity (μmol h^−1^ g^−1^) of PET‐hydrolysing lipases against benchmark lipase substrate Tri‐C10 and PET substrates.

Enzyme	Specific activity (μmol h^−1^ g^−1^)				
	Tri‐C10^a^	nPET^b^		pPET^b^	
	40°C	40°C	60°C	40°C	60°C
Lip_Bv_	1499 ± 4	943 ± 10	2706 ± 12	255 ± 22	108 ± 18
Lip_Sh1_	1180 ± 9	431 ± 33	0^c^	0^c^	0^c^
Lip_MRD9_	1275 ± 30	3116 ± 53	2255 ± 19	240 ± 13	71 ± 5
Lip_Sh2_	1065 ± 22	48 ± 9	0^c^	0^c^	0^c^
Lip_Sh3_	1032 ± 11	18 ± 2	0^c^	0^c^	0^c^

^a^ Experimental conditions for Tri‐C10 assays: [Substrate], 24 mM from stock solutions prepared in DMSO; [Enzyme], 0.14 mg mL^−1^; buffer, 40 mM HEPES containing 3 mM CaCl_2_, pH 7.0; temperature, 40°C; shaking, 950 rpm; reaction time, 24 h.

^b^ Experimental conditions for PET hydrolysis assays with nPET and pPET using Lip_Bv_ and Lip_MRD9_: [Substrate], 1.55 mg mL^−1^ nPET or 7 mg mL^−1^ pPET; [Enzyme], 0.5 mg mL^−1^; buffer, 40 mM HEPES containing 3 mM CaCl_2_, pH 7.0; temperature, 40°C or 60°C; reaction time, 2 h.

^c^ Experimental conditions for PET hydrolysis assays with nPET and pPET using Lip_Sh1_, Lip_Sh2_ and Lip_Sh3_: [Substrate], 1.65 mg mL^−1^ nPET or 7 mg mL^−1^ pPET; [Enzyme], 1 mg mL^−1^; buffer, 40 mM HEPES containing 3 mM CaCl_2_, pH 7.0; temperature, 40°C or 60°C; reaction time, 6 h.

To place the activities of the newly identified PET‐hydrolysing lipases into context, representative engineered PET hydrolases were included as benchmarks (Table 5). LCC‐ICCG, LCC‐WCCG and HotPETase were evaluated alongside Lip_Bv_ under identical experimental conditions using the same commercial PET powder, allowing direct comparison of their activities. Table 5 also summarizes representative activities reported in the literature, although these should be interpreted with caution because they were obtained using different PET substrates and experimental conditions. At 40°C, Lip_Bv_ exhibited activity within the same order of magnitude as the engineered benchmark enzymes. In contrast, at 60°C the engineered PET hydrolases were approximately 14‐ to 17‐fold more active than Lip_Bv_, consistent with their extensive optimization through protein engineering for high‐temperature PET depolymerization. Nevertheless, Lip_Bv_ remained active against highly crystalline PET powder at both temperatures, highlighting its intrinsic PET‐hydrolysing capability as a naturally occurring true lipase and supporting the ability of the PELE‐based workflow to identify promising candidates for future protein engineering efforts.

**TABLE 5:** PET powder hydrolysis activities of representative engineered PET hydrolases.

Enzyme	Family	T (°C)	Substrate	Specific activity (μmol h^−1^ mg^−1^)	References
LCC‐ICCG	Cutinase	40	pPET^a^	116 ± 20	This work
LCC‐WCCG	Cutinase		pPET^a^	492 ± 19	This work
Hot‐PETase	PETase		pPET^a^	583 ± 14	This work
LCC‐ICCG	Cutinase	50	pPET^a^	2795 ± 56	This work
LCC‐WCCG	Cutinase		pPET^a^	1992 ± 27	This work
Hot‐PETase	PETase		pPET^a^	1722 ± 15	This work
LCC‐ICCG	Cutinase	60	pPET^a^	1517 ± 6	This work
LCC‐WCCG	Cutinase		pPET^a^	1857 ± 29	This work
Hot‐PETase	PETase		pPET^a^	1534 ± 21	This work
FAST‐PETase	PETase	50	Gf‐pPET^b^	348	Arnal et al. (2023)
HotPETase	PETase	60	Gf‐pPET^b^	≈1100	Arnal et al. (2023)
PES‐H1L92F/Q94Y	Cutinase	65	Gf‐pPET^b^	481	Arnal et al. (2023)
LCC‐ICCG	Cutinase	68	Gf‐pPET^b^	963	Arnal et al. (2023)

^a^ Hydrolysis rate determined using commercial Goodfellow PET powder (Gf‐pPET; Goodfellow Cambridge; ref. ES30PD000132) (> 40% crystallinity and < 300 μm) using the same conditions described in table 4.

^b^ Hydrolysis rate reported by Arnal et al. (2023), obtained under experimental conditions different from those used in this study, using cryo‐ground amorphous Goodfellow PET (Gf‐PET; particle size < 500 μm; crystallinity 7.7%) prepared from PET sheets, as well as post‐consumer waste PET powder (PcW‐PET; crystallinity 14.6%).

**FIGURE 3:**
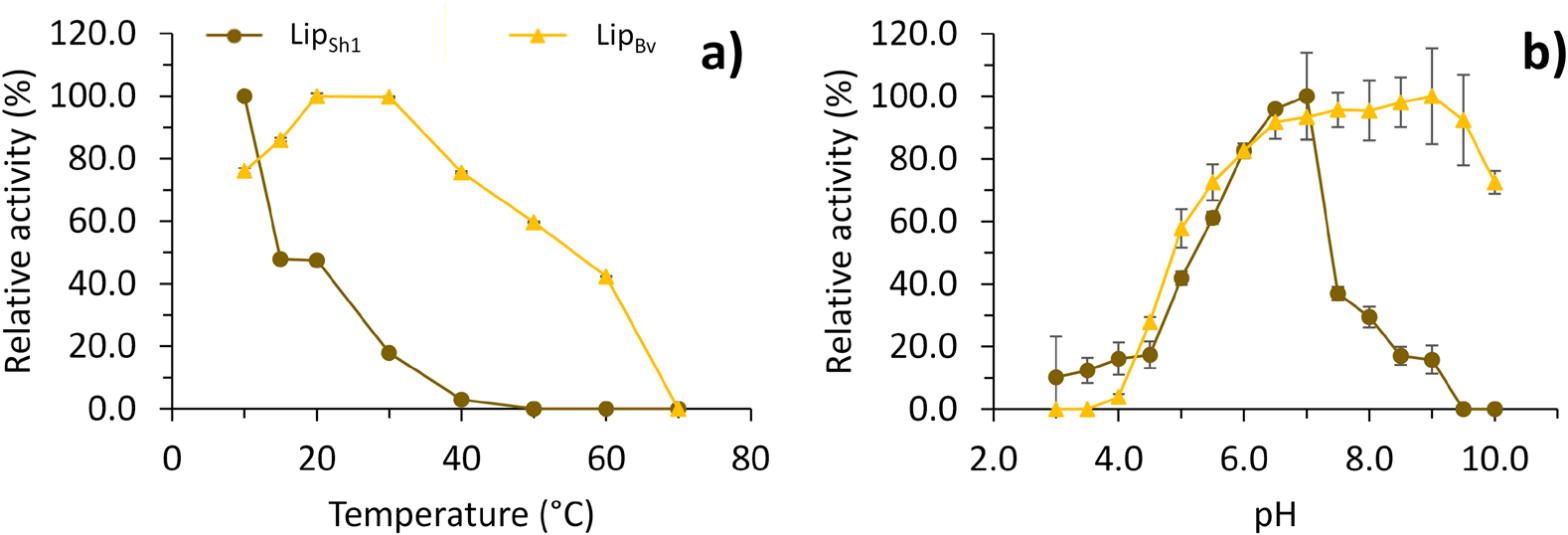
Physicochemical characterization of Lip_Sh1_ and Lip_Bv_, which display both the ability to hydrolyse triglycerides and PET substrates. (a) Temperature activity profile using Tri‐C8 as the substrate and a NEFA assay kit. (b) pH activity profile determined at 40°C using *p*NPB as the substrate. In all cases, the data are presented as the means ± standard deviations (SDs) of triplicate measurements (*n* = 3). Standard deviations were calculated using the STDEV.S function in Excel 2019. Additional experimental details are provided in the Methods section.

Lip_Sh1_ and Lip_Bv_ were subsequently analysed to better understand the observed differences in PET hydrolysis. Temperature profiling revealed marked differences (Figure 3a). Lip_Sh1_ exhibited maximal activity at 10°C and low activity above 30°C–40°C, which is consistent with its limited PET hydrolysis at elevated temperatures. In contrast, Lip_Bv_ showed optimal activity at 20°C–30°C while retaining substantial activity up to 60°C, indicating greater thermal robustness. Both enzymes were active across a broad range of pH values but displayed distinct profiles (Figure 3b). Lip_Bv_ showed maximal activity under slightly alkaline conditions (pH 8.0–9.0) and retained activity up to a pH of 11.0, indicating broad pH stability under alkaline conditions. In contrast, Lip_Sh1_ was most active between pH 5.5 and 7.0, retained activity at acidic pH values (3.0–4.0), but rapidly lost activity above pH 7.5.

### 3.3 PET‐Hydrolysing Lipases Form Clusters Distinct From Those of Other Extremophilic and Known PETases

**FIGURE 4:**
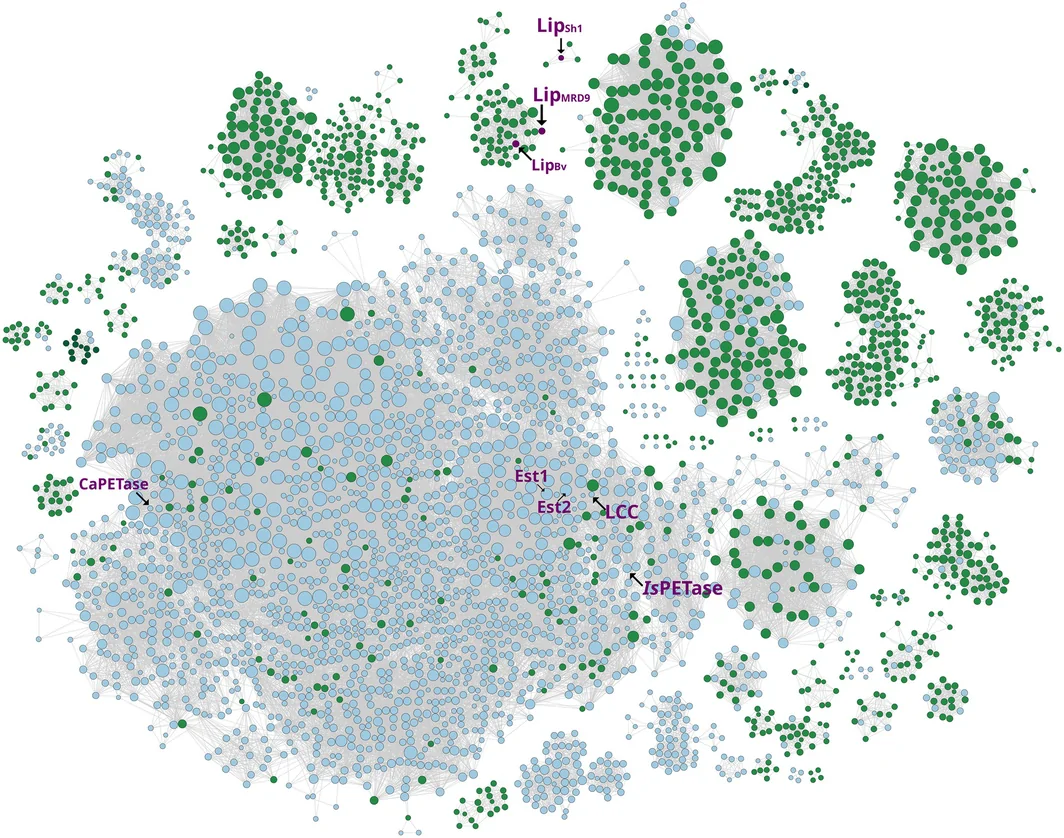
SSN analysis of candidate PETases and previously reported PET‐hydrolysing enzymes. Node size is proportional to node degree. Lip_MRD9_, Lip_Bv_ and Lip_Sh1_ are highlighted in purple. Light green nodes correspond to bacterial sequences, whereas dark green nodes correspond to archaeal sequences. Light blue nodes represent reference PETases, including sequences previously reported by Seo et al. (2025).

Having identified Lip_Sh1_ and Lip_Bv_ as enzymes capable of hydrolysing both lipid substrates and PET under the assay conditions used in this study, we next searched the same extremophile‐derived genome dataset for classical PETase homologue sequences and analysed them alongside previously characterized PETases of non‐extremophilic origin. The aim was to place these enzymes within the broader PETase sequence landscape and determine whether their dual catalytic behaviour is linked to distinctive evolutionary or structural features. A homology‐based search against a reference dataset of characterized PETases (Table S2a) revealed 1322 candidate protein sequences encoding putative PETases (Table S2b) from the 18,082 extremophile genomes retrieved from the GTDB (Table S1b). To examine the relationships between these candidate PETases, the dual‐function enzymes Lip_Sh1_ and Lip_Bv_, and previously described PET‐hydrolysing enzymes (Table S2c), SSN analysis was performed following the methodology described by Seo et al. (2025) (Figure 4). The resulting network comprised 70 clusters containing 45,845 edges and 40 nodes, revealing that most PETase candidates originating from extremophilic organisms segregated from previously mapped PETases, highlighting the evolutionary divergence of these newly identified enzymes. Moreover, the Lip_Bv_ cluster contained 48 proteins, whereas the Lip_Sh1_ cluster contained only four sequences (Table S3). Notably, neither cluster contained classical PETases from our reference dataset, indicating that Lip_Bv_‐ and Lip_Sh1_‐like enzymes occupy distinct regions of the PETase landscape.

**FIGURE 5:**
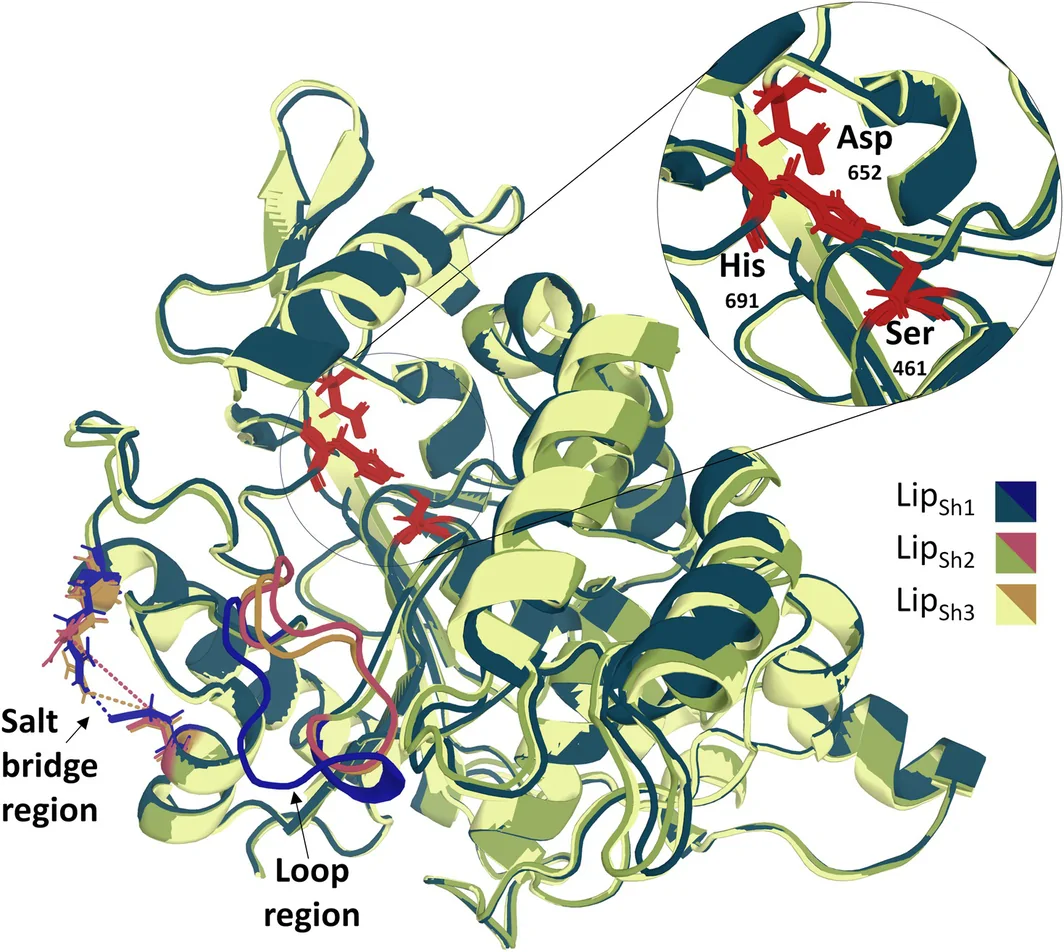
Structural alignment of proteins in the Lip_Sh1_ cluster. Initial structure predictions revealed low confidence scores for the C‐terminal regions of both proteins. InterProScan analysis consistently identified intrinsically disordered regions spanning approximately the first half of the sequences; therefore, only the α/β‐hydrolase catalytic domain was retained for structural analyses. Aligned structural models of Lip_Sh1_, Lip_Sh2_ and Lip_Sh3_ are shown. The catalytic triads are highlighted in firebrick, and a salt bridge and a variable loop region located near the active site are indicated.

Based on the SSN topology, we next evaluated whether the sequences clustering with Lip_Sh1_ or Lip_Bv_ also hydrolysed both lipid substrates and PET under our assay conditions. Because the Lip_Bv_ cluster includes the previously characterized lipase‐PETase Lip_MRD9_ (Vidal et al. 2024), proteins within this group were expected to share similar catalytic properties. We therefore focused on the smaller and distinct Lip_Sh1_ cluster, which contained only four closely related sequences that were potentially novel PET‐hydrolysing lipases. After removing redundant entries, two homologous proteins, TRL33698 and TRL79618, were selected for further characterization and renamed Lip_Sh2_ and Lip_Sh3_, respectively. Both enzymes shared high sequence identity with Lip_Sh1_ (~81%) and originated from *Staphylococcus hominis* isolates recovered from the Sevilleta Desert (New Mexico, USA), the same environment as Lip_Sh1_ (Table S1e). Consistent with this high degree of homology, structural alignment revealed highly similar architectures among the three proteins (RMSDs = 0.265 relative to Lip_Sh1_), particularly around the catalytic triad (Figure 5). Minor differences were detected in the loop near the active site, which potentially affect local flexibility and catalytic efficiency. The canonical Ser‐His‐Asp catalytic triad and its protonation state were conserved in the three proteins.

Further experimental validation confirmed that both enzymes exhibited lipase activity when Tri‐C10 was used as a substrate. Their activities were comparable to that of Lip_Sh1_, albeit slightly lower (Lip_Sh2_: 1065 ± 22 μmol NEFA h^−1^ g^−1^; Lip_Sh3_: 1032 ± 105 μmol NEFA h^−1^ g^−1^). Similarly, PET hydrolysis assays using nPET confirmed that compared with Lip_Sh1_, both enzymes retained PET‐hydrolysing activity, albeit with lower catalytic efficiency (Table 4). Lip_Sh1_ displayed the highest activity (431 ± 3 μmol h^−1^ g^−1^), whereas the activities of Lip_Sh2_ and Lip_Sh3_ were lower, at 48 ± 9 and 18 ± 2 μmol h^−1^ g^−1^, respectively. These results show that enzymes clustered with Lip_Sh1_ can hydrolyse both lipidic and PET substrates, supporting the hypothesis that this SSN cluster represents a previously uncharacterized group of PET‐active lipases.

### 3.4 Structural Determinants Underlying the Hydrolysis of Both Lipid Substrates and PET

The identification of Lip_Sh1_ and its homologues (Lip_Sh2_ and Lip_Sh3_) as true lipases capable of hydrolysing both lipids and PET under the assay conditions used in this study prompted us to investigate whether specific structural features could explain this catalytic behaviour compared with Lip_Sa_ and Lip_Sha_, which exhibited lipolytic activity but no detectable PET hydrolysis (Table 3) despite belonging to the same structural group of Family I.IV lipases (Figure 1). Notably, Lip_Sa_ showed productive substrate binding during the computational simulations, whereas Lip_Sha_ did not, suggesting that subtle variations within the active site may influence PET accommodation and hydrolysis efficiency compared with that of lipid esters.

**FIGURE 6:**
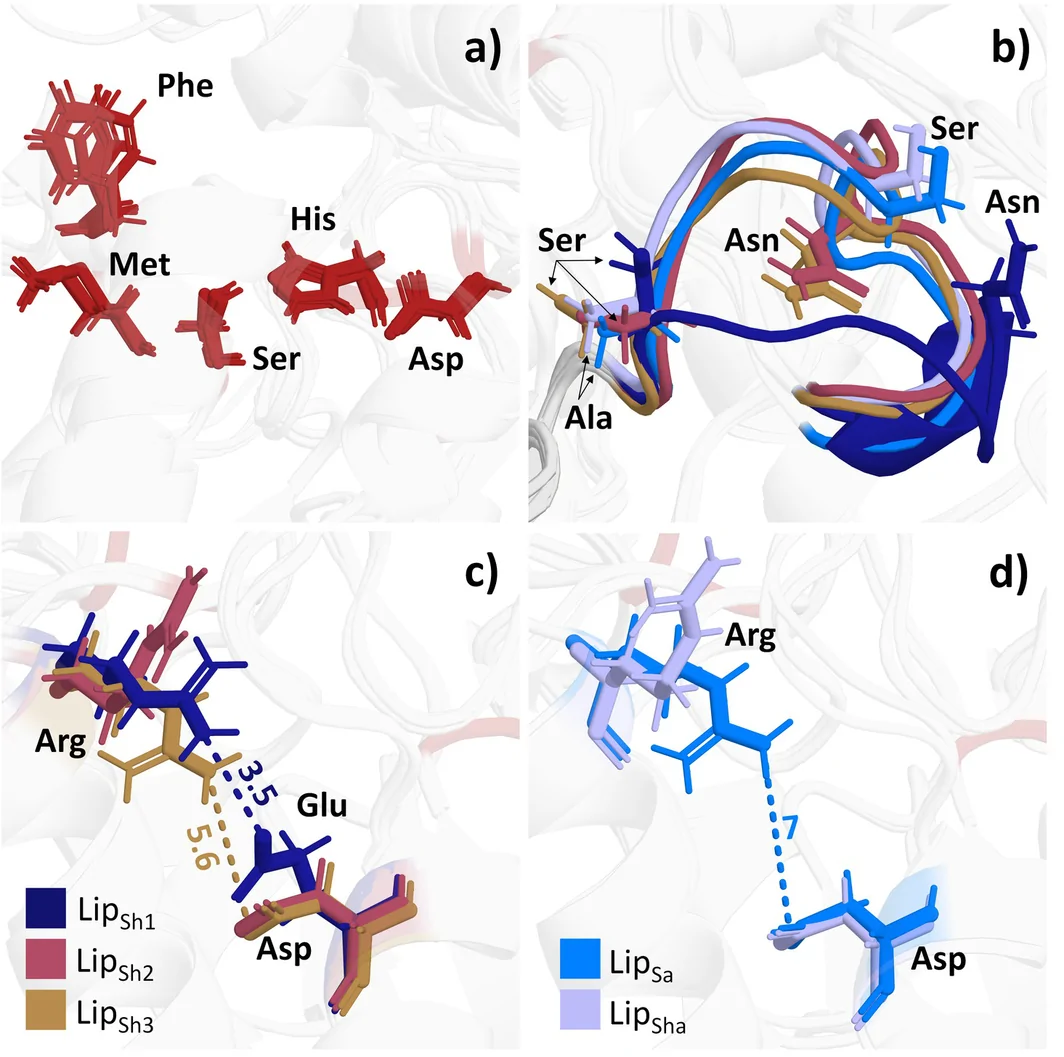
Structural determinants associated with PET‐hydrolysing activity in consensus disordered proteins. (a) Magnified view of the catalytic region showing the superposition of key residues involved in PET hydrolysis, including the catalytic triad and the oxyanion hole highlighted in firebrick. The high structural conservation of these residues across proteins indicates that activity differences are unlikely to have arisen from changes in the catalytic machinery itself. (b) Structural alignment highlighting residue differences in the loop near the active site. Active enzymes (Lip_Sh1_, Lip_Sh2_ and Lip_Sh3_) contain an asparagine and a serine residue, whereas inactive proteins (Lip_Sa_ and Lip_Sha_) contain a serine and an alanine residue, potentially altering the local electrostatic environment near the pocket entrance. (c) Structural representation of the putative salt bridge connecting the N‐terminal and C‐terminal helices in active enzymes. In Lip_Sh1_, a glutamic acid residue interacts with a conserved arginine, potentially stabilizing the local structure around the loop region more easily than the putative salt bridge in Lip_Sh2_ and Lip_Sh3_, which instead contain aspartic acid. (d) The equivalent region in inactive proteins exhibits a greater distance between charged residues and increased variability in terms of side chain orientation, suggesting a weaker or transient interaction that reduces the structural stability of the loop region.

To explore the molecular basis of this functional divergence, we performed a comparative structural analysis focusing on the active site architecture, substrate‐binding pocket geometry and neighbouring loop regions potentially involved in PET binding and hydrolysis. First, we performed a comparative analysis of the structural models of the 5 lipases with the Family I.IV fold, namely, Lip_Sha_, Lip_Sa_, Lip_Sh1_, Lip_Sh2_ and Lip_Sh3_ (Figure 1). Multiple structural alignment (MSTA) was carried out using FoldMason (Gilchrist et al. 2026) with up to five refinement iterations, and the resulting alignments were manually inspected to identify structural features potentially associated with PET hydrolysis activity. Structural alignment (Figure S6) and the corresponding guide tree (Figure S7) revealed early divergence of Lip_Sh1_ and its homologues, Lip_Sh2_ and Lip_Sh3_, relative to the remaining sequences. Further inspection of the alignment (Figure S8) and structural comparisons revealed a notable difference in the loop region located near the entrance of the main catalytic pocket (Figure 5). In Lip_Sh1_, this loop appears more open and positioned farther from the pocket entrance than the corresponding region in the other proteins does. Importantly, the catalytic residues, including the catalytic triad and oxyanion hole, are structurally conserved and superimpose well across all the proteins (Figure 6a), indicating that the differences in the ability to hydrolyse PET substrates are unlikely to have arisen from structural variations affecting substrate accessibility to the active site. Given the structural differences observed near the pocket entrance, we next performed a sequence alignment of this region. Consistent variation between PET‐hydrolysing and non‐PET‐hydrolysing Family I.IV lipases was identified at alignment positions 23 and 26. In the PET‐hydrolysing enzymes (Lip_Sh1_, Lip_Sh2_ and Lip_Sh3_), these positions are occupied by asparagine and serine residues, respectively, whereas the non‐PET‐hydrolysing proteins (Lip_Sa_ and Lip_Sha_) contain serine and alanine residues, respectively (Figure 2c, Figure 6b, Figure S8). Structural inspection revealed that the side chain orientations at these positions were conserved across proteins; however, their chemical properties differed. In non‐PET‐hydrolysing enzymes, the serine residue contributes to a more negatively charged local environment, whereas the asparagine present in PET‐hydrolysing enzymes may create a more favourable electrostatic environment for substrate accommodation.

An additional structural difference was identified as a potential salt bridge connecting the first and last helices of the protein. In Lip_Sh1_, alignment position 39 corresponds to a glutamic acid residue that interacts with a conserved arginine (361) located in the C‐terminal helix at a distance of approximately 3.5 Å (Figure 6c), which is consistent with a stabilizing salt bridge. In the Lip_Sh1_ homologues, the corresponding residue is an aspartic acid, increasing the distance between the charged residues to approximately 5.6 Å. A similar arrangement was observed in the non‐PET‐hydrolysing proteins Lip_Sa_ and Lip_Sha_ (Figure 6d), suggesting that salt bridge formation in these enzymes may depend on transient side chain conformations.

These structural differences were consistent with the distinct PET degradation profiles revealed by mass spectrometry analyses (see Methods) of the reaction products obtained with the different PET‐hydrolysing lipases (Figure S9). Consistent with these data, Lip_MRD9_ generated a broad spectrum of PET‐derived intermediates, including TETETE, TETETET, TETETETE and TETETETETE (Figure S10). A similar degradation profile was observed for Lip_Bv_ (Figure S11) and Lip_Sh1_ (Figure S12), both of which also generated long PET‐derived oligomers, suggesting an increased capacity to accommodate and process extended PET fragments. In contrast, Lip_Sh2_ generated mainly TET as the primary degradation product (Figure S13), whereas Lip_Sh3_ accumulated predominantly shorter oligomers such as TET, TETE and ETETE (Figure S14). The shared degradation patterns of Lip_MRD9_, Lip_Bv_ and Lip_Sh1_ are consistent with their more open groove‐like active‐site architecture lacking a canonical LID domain.

## 4 Discussion

The discovery of enzymes capable of depolymerizing PET has significantly expanded over the past decade (Pérez‐García et al. 2025), yet the structural diversity of known PET hydrolases remains largely concentrated within the polyesterase–cutinase branch of the α/β‐hydrolase superfamily (Austin et al. 2018; Sui et al. 2023; Vongsouthi et al. 2025). The existence of few lipases with PET‐hydrolysing activity (Herzog et al. 2006; Wang et al. 2008; Eberl et al. 2009; Vidal et al. 2024) suggests that this function may not be exclusive to canonical PETases, but whether true lipases represent a broader reservoir of PET‐hydrolysing enzymes has remained largely unexplored.

Canonical cutinases typically possess catalytic residues located within a shallow and surface‐exposed cleft that facilitates the accommodation of bulky terephthalate‐containing substrates with limited steric hindrance (Tournier et al. 2020), together with an oxyanion hole that stabilizes the tetrahedral transition state during catalysis (Austin et al. 2018). In contrast, many lipases contain LID domains and deeper, more hydrophobic catalytic pockets that may restrict the access of PET chains. This structural organization likely explains why only a subset of lipases display PET‐hydrolysing activity. Previous studies have also shown that subtle variations in active site architecture, substrate‐binding cleft geometry and surrounding hydrophobic surfaces strongly influence PET recognition and hydrolysis (Austin et al. 2018; Berselli et al. 2021; Liu et al. 2023). In particular, aromatic and hydrophobic residues surrounding the catalytic cleft have been proposed to facilitate interactions with terephthalate moieties (Austin et al. 2018; Vidal et al. 2024).

In this context, our results indicate that PET‐active lipases may constitute structurally distinct catalytic solutions for PET hydrolysis. The lipases identified here are characterized by relatively exposed catalytic sites and, in several cases, the absence of a canonical LID domain, resembling other lid‐less lipases such as CalB, which has also been associated with PET oligomer hydrolysis (Joo et al. 2018). Structural analyses of the Lip_Sh1_ cluster suggested that PET‐hydrolysing activity is not determined by differences in the catalytic machinery itself, which remains highly conserved, but rather by structural features modulating substrate accessibility. In particular, the conformations of the loops surrounding the catalytic pocket appear to influence cleft openness and the accommodation of PET‐derived oligomers, a feature previously recognized as a key determinant of PETase efficiency (Wei and Zimmermann 2017a, 2017b; Duan et al. 2023). In the Family I.IV lipases identified here, subtle variations near the pocket entrance, including differences in the local electrostatic environment and the presence of a stabilizing salt bridge, may contribute to maintaining a more accessible catalytic cleft. Considering that PET oligomers contain negatively charged carbonyl oxygens, the presence of asparagine near the pocket entrance may facilitate substrate accommodation by reducing unfavourable electrostatic interactions during binding. Likewise, the glutamic acid residue present in Lip_Sh1_ may promote the formation of a more stable salt bridge than the corresponding aspartic acid found in the related Family I.VI lipases. The longer side chain of glutamic acid could bring neighbouring structural elements into closer proximity and influence the positioning of the adjacent loop region. Such an arrangement may help maintain a more open catalytic cleft and thereby facilitate substrate access. Similar structural features may also explain the distinct lipolytic profiles observed among the Family I.VI enzymes. In particular, the more accessible and polar environment predicted near the Lip_Sh1_ catalytic pocket could favour the accommodation of medium‐chain triglycerides and alkyl esters, whereas the narrower and more hydrophobic pockets of Lip_Se_, Lip_Sa_ and Lip_Sha_ may preferentially support the binding of shorter hydrophobic lipid substrates. The correlation detected between active site accessibility and degradation product distribution further supports this interpretation, as enzymes displaying more open catalytic architectures generated longer PET‐derived oligomers. In this context, Lip_Sh1_ appears to adopt an intermediate active site configuration capable of accommodating extended PET fragments, whereas the more open groove‐like architectures observed in Lip_Bv_ and Lip_MRD9_, together with the absence of a canonical LID domain, may further facilitate the binding and processing of longer PET chains. Collectively, these observations support the idea that subtle differences in active‐site topology, loop organization and substrate channel accessibility strongly influence substrate accommodation, degradation efficiency and product distribution in PET‐active true lipases.

Here, we demonstrate that extremophilic microorganisms constitute a previously unexplored reservoir of evolutionarily distinct PET‐hydrolysing true lipases, expanding the known diversity of PET‐hydrolysing enzymes beyond classical PETases. Among the six enzymes experimentally characterized in this study (Lip_Bv_, Lip_Se_, Lip_Sa_, Lip_Sh1_, Lip_Sha_ and Lip_Re_), all were confirmed to hydrolyse lipid substrates, supporting the robustness of the computational pipeline for identifying bona fide true lipases. PET‐hydrolysing activity, however, was detected only for Lip_Bv_ and Lip_Sh1_, indicating that PET hydrolysis represents a more restricted functional trait that cannot be inferred solely from lipase activity or sequence similarity. Importantly, one of the evolutionary lineages identified in this study is not represented solely by Lip_Bv_. The corresponding cluster comprises 48 hydrolases, including Lip_Bv_ and the previously characterized lipase–PETase Lip_MRD9_ (Vidal et al. 2024), which share 75.35% sequence identity. The remaining members of the cluster display sequence identities ranging from 64.1% to 95.5% relative to these experimentally validated enzymes. Although these observations suggest that this lineage represents a promising reservoir of PET‐hydrolysing true lipases, additional homologous enzymes will need to be experimentally characterized before determining the extent to which PET‐hydrolysing activity is conserved across the lineage. Therefore, the lineages identified in this study should be regarded as promising reservoirs of PET‐active true lipases rather than evidence that PET hydrolysis is a universal property of all lineage members.

To further place these lineages into an evolutionary context, we reconstructed a phylogenetic tree including representative PETases, cutinases, esterases and true lipases together with the PET‐hydrolysing lipases identified in this study (Figure S15). The phylogenetic analysis was fully consistent with the SSN, with the newly identified PET‐hydrolysing true lipases forming distinct lipase lineages clearly separated from canonical PETases such as IsPETase, FAST‐PETase and LCC. Moreover, the PET‐active homologues identified in this work clustered together within their respective lipase families, supporting the existence of previously unrecognized PET‐hydrolysing true lipase lineages rather than isolated functional variants. Together, the SSN and phylogenetic analyses support that PET‐hydrolysing activity has emerged independently in multiple α/β‐hydrolase lineages.

These findings extend the findings of previous sequence landscape studies focused on polyesterases, where PET‐hydrolysing activity was found to be concentrated in defined regions of sequence space (Seo et al. 2025; Seo and Wei 2025), and suggest that PET‐hydrolysing functional islands may also exist within lipase families. Interestingly, unlike many canonical cutinases and PETases that contain conserved disulphide bonds that contribute to structural stabilization (Joo et al. 2018), PET‐active lipases identified here lack disulphide bonds within their catalytic scaffold. Together with the inherent robustness and industrial relevance commonly associated with lipases (Tournier et al. 2020), these features suggest that PET‐hydrolysing true lipases may represent a complementary class of biocatalysts for polyester depolymerization applications. The use of both nPET and higher‐crystallinity PET powder, a standard substrate for the biochemical characterization of PET hydrolases (Erickson et al. 2022), enabled enzyme activity to be evaluated on substrates with different levels of accessibility, while future studies using bottle‐grade PET or commercial PET films will be important to assess industrial relevance. Although Lip_Bv_ and Lip_Sh1_ do not match the catalytic performance of the most efficient engineered PETases, the objective of this study was to expand the diversity of PET‐active enzymes rather than identify superior catalysts. Their distinct structural features provide alternative scaffolds for future protein engineering and broaden the repertoire of enzymes available for PET biodegradation and recycling.

Beyond the identification of PET‐active true lipases and the structural determinants associated with their dual catalytic behaviour, this work also provides a broader view of the diversity of PETases in extremophilic microorganisms. Although PETases from thermophiles and other extremophiles have previously attracted attention because of their increased stability and potential industrial applicability (Wei and Zimmermann 2017a, 2017b; Gargano et al. 2025; Hu et al. 2025), the large‐scale exploration of PETase diversity across extremophile genomes has remained limited. The broad diversity of PETase homologue sequences identified here, many of which clustered separately from previously characterized PETases, suggests that extremophilic microorganisms harbour a substantially wider repertoire of PET‐hydrolysing enzymes than currently represented by experimentally characterized PETases. Although elucidating the evolutionary origin of PET‐hydrolysing activity was beyond the scope of this work, the observed distribution of PET‐active lipases is consistent with the idea that this activity did not evolve in response to PET itself. Because PET is a synthetic polymer, PET hydrolysis is more likely to represent a promiscuous activity of enzymes that primarily function on natural ester‐containing substrates. This interpretation is consistent with the observation that the enzymes characterized in this study retain substantial lipolytic activity towards natural lipid substrates (Tables 3 and 4) while belonging to established true lipase families rather than canonical PETase lineages. Similar observations have recently been reported for other α/β‐hydrolases, including a feruloyl esterase with PET‐hydrolysing activity (Mamtimin et al. 2024).

More broadly, this work illustrates the potential of integrating genome mining, structural modelling and enzyme–substrate simulations to accelerate the discovery of enzymes capable of degrading synthetic polymers. Recent studies have demonstrated that structure‐guided computational screening can substantially reduce the experimental search space when large sequence datasets are explored (Guo et al. 2023; Markus et al. 2023), and the agreement observed between computational predictions and experimental validation in this study further supports the robustness of this strategy. The computational workflow employed the soluble PET oligomer ETETETETE as a model substrate rather than crystalline PET surfaces. Although this provides a well‐defined system for identifying productive binding pockets and catalytically relevant enzyme–substrate conformations, it does not fully reproduce the structural complexity and accessibility of polymeric PET (Robles‐Martín et al. 2023). Consequently, the simulations were intended to prioritize candidate enzymes with the potential to recognize PET ester bonds rather than to predict quantitative PET depolymerization. Moreover, enzyme‐substrate simulations are sensitive to potential errors in the generated structure, in the lack of an extensive exploration of the conformational landscape and in the approximations in substrate and receptor energy functions. These simulations should not be seen as a ground truth but as an efficient path for enrichment, narrowing down the list for experimental test and increasing the number of true positives. Collectively, our findings suggest that true lipases represent a previously underappreciated group of PET‐hydrolysing enzymes and that expanding the search beyond canonical PETases may reveal additional catalytic scaffolds capable of contributing to biological polyester recycling. Furthermore, applying the integrated pipeline developed here to explore the structural dynamics of lipase catalytic pockets and the broader lipase sequence space may enable the discovery of a new generation of PET hydrolases.

## 5 Conclusions

In this study, we combined genome mining of extremophilic microorganisms with structural modelling, enzyme–substrate simulations and experimental validation to expand the known landscape of PET‐hydrolysing enzymes. Our results reveal that extremophile genomes harbour not only diverse PETase homologues but also previously unrecognized groups of true lipases capable of hydrolysing both lipid substrates and PET under the assay conditions used in this study. Beyond the identification of new PET‐active enzymes, this work highlights the potential of integrating large‐scale computational screening and structure‐guided analyses to explore unexplored regions of the enzyme sequence space and prioritize functional candidates from large genomic datasets. Expanding these approaches to broader microbial diversity and lipase families may accelerate the discovery of robust PET hydrolases and novel catalytic scaffolds for sustainable polyester biodegradation and recycling.

## Author Contributions

**Manuel Ferrer:** conceptualization, methodology, resources, writing – review and editing, writing – original draft, funding acquisition, investigation, supervision, project administration. **Ana Robles‐Martín:** methodology, data curation, investigation, formal analysis. **Rafael Bargiela:** project administration, funding acquisition, writing – review and editing, writing – original draft, conceptualization, investigation, methodology, data curation, formal analysis, supervision, resources. **Laura Fernandez‐Lopez:** methodology, formal analysis, validation, investigation. **José M. González‐Romero:** methodology, data curation, investigation, formal analysis, resources, visualization, writing – original draft. **Paula Vidal:** methodology, investigation, validation, formal analysis. **Francisco J. Plou:** writing – review and editing, methodology, resources, supervision, funding acquisition, project administration. **Rubén Muñoz‐Tafalla:** methodology, investigation, visualization, data curation, formal analysis, resources, writing – original draft. **José L. Gonzalez‐Alfonso:** methodology, validation, formal analysis, investigation. **Víctor Guallar:** conceptualization, methodology, resources, project administration, supervision, writing – review and editing, writing – original draft. **David Almendral:** methodology, investigation, validation, formal analysis.

## Funding

This work was supported by European Commission, No. 101000327 (FuturEnzyme), No. 101060625 (Nymphe). Agencia Estatal de Investigación, PID2023‐153370OB‐100, PID2022‐136367OB‐C31, TED2021‐130544B‐I00. Consejería de Educación, Ciencia y Universidades of the Comunidad de Madrid, 2024‐T1/ECO‐31227.

## Conflicts of Interest

The authors declare no conflicts of interest.

## Supporting information


**Table S1:** Reference and candidate true lipases identified during the genome mining workflow. The table summarizes the datasets used for the identification of candidate true lipases from extremophilic genomes. Reference lipase sequences were compiled and employed as query sequences for similarity searches against the GTDB and additional protein databases. (a) Reference dataset of true lipases compiled to be used as reference queries for BLAST searches. (b) List of genomes retrieved from the GTDB selected as possible extremophiles to search lipases. (c) Initial list of selected sequences from extremophiles with at least 60% of sequence identity and minimum alignment length of 100 residues with the reference lipases. (d) Filtered list of 50 presumptive true lipases identified, after signal peptide detection and reducing sequence redundancy, further preselected candidates for structural and functional analysis. Metadata and sequence features of candidate enzymes identified in this study, including GTDB genome IDs, UniProt matches, sequence identity, signal peptide prediction, sequences used for gene synthesis, environmental origin, taxonomic classification, catalytic triad residues and predicted structural features. (e) Final selected candidates after structural and functional analysis. Sequence features of candidate enzymes identified in this study, including GTDB genome IDs, UniProt matches, sequence identity (%) and alignment length. It shows original and synthetic protein sequences (signal peptides in blue, SignalP‐5.0), pET‐45(+) plasmid constructs synthesized by GenScript Biotech (EG Rijswijk, The Netherlands; ref. U063CUMTG0, U790YVJEG0 and U5150FMPG0), antibiotic resistance (ampicillin), inducer (IPTG), molecular weights (calculated with ExPASy; https://web.expasy.org/compute_pi/), catalytic triad residues, predicted structural features, environmental origin and extremophily and taxonomic classification. Due to the size and complexity of the dataset, Table S1 is provided as a separate Microsoft Excel (.xlsx) file, stored and accessible through the following Zenodo link: https://doi.org/10.5281/zenodo.20524880. All worksheets are labelled according to the workflow stages described in the manuscript and can be accessed using standard spreadsheet software.

**Table S2:** Reference PETases and candidate PETase homologue sequences identified from extremophilic genomes. This table summarizes the datasets used for the identification of putative PETases from extremophilic microbial genomes. Experimentally characterized PETase sequences were compiled and employed as query sequences for homology‐based searches against the GTDB and other protein databases. (a) Reference dataset of experimentally validated PETases used as queries for similarity searches. (b) Set of 1322 candidate PETase homologue sequences identified from 18,082 extremophilic microbial genomes retrieved from the GTDB. Metadata and sequence features are provided for each candidate, including GTDB genome ID, UniProt matches, sequence identity, taxonomic classification and environmental origin. (c) List of sequences for SSN. Owing to the large size of the dataset, Table S2 is provided as a separate Microsoft Excel (.xlsx) file, stored and accessible through the following Zenodo link: https://doi.org/10.5281/zenodo.20524880. The file contains multiple worksheets corresponding to the different stages of the PETase mining workflow and can be viewed using standard spreadsheet software.

**Table S3:** Clustal Omega pairwise distances of proteins belonging to the Lip_Bv_ and Lip_Sh1_ SSN clusters relative to their reference sequences.
**Figure S1:** Best docking poses of candidate Lip_Se_, which showed the highest CBFE for both substrates. (a) Docked PET tetramer and (b) docked Tri‐C10. In both panels, the protein is shown as a yellow cartoon, highlighting the catalytic triad residues and the substrate represented in stick format. Hydrogen‐bond interactions among the catalytic triad residues are shown in pink, while the distance between the catalytic serine and the closest ester carbon of the substrate is indicated in cyan. These representations illustrate the catalytic poses selected for the PELE simulations, showing the reactive substrate atoms positioned near the catalytic serine and providing a visual example of a productive binding mode for each substrate.
**Figure S2:** Energy interactions of candidate enzymes. (a) Interaction energy profiles for the 23 candidate enzymes using Tri‐C10 as the substrate. Each scatter plot shows all poses sampled during the simulation for a given protein. The *x*‐axis represents the distance between the reactive oxygen atom of the catalytic serine and the closest ester bond of the triglyceride ligand (Å), while the *y*‐axis shows the interaction energy (kcal mol^−1^). The colour scale indicates the total energy of each pose (kcal mol^−1^). (b) Ranking based on the calculated CBFE. The black arrow indicates the selected candidates for the next stage.
**Figure S3:** PELE simulation results obtained using the PET tetramer as the ligand. Scatter plots show all poses sampled throughout the simulations for each protein. The *x*‐axis indicates the distance between the reactive oxygen atom of the catalytic serine and the closest non‐terminal ester bond of the polymeric ligand (Å), while the *y*‐axis shows the interaction energy (kcal mol^−1^). The colour scale indicates the total energy of each pose (kcal mol^−1^). (a) Interaction energy profiles for the three benchmark PETases: Fast‐PETase, HiC and LCC‐ICCG. (b) Interaction energy profiles for the 15 selected candidate enzymes. (c) Ranking based on the calculated Catalytic Binding Free Energy (CBFE). The black arrow indicates the highest CBFE value among the benchmark PETases; therefore, only candidates with lower CBFE values were selected for experimental validation.
**Figure S4:** SDS–PAGE analysis of recombinant enzymes. 12% polyacrylamide SDS PAGE of enzymes. Lane 1: total proteins. Lane 2: soluble protein. Lane 3: pure proteins 10 μg.
**Figure S5:** Structural comparison of the active sites of Lip_Bv_ and Lip_Sh1_. Surface representations of the Alphafold predicted structures highlighting the active‐site residues (coloured). Front and side/cross‐sectional views illustrate the distinct active‐site architectures of the two enzymes, with Lip_Bv_ displaying an open surface groove and Lip_Sh1_ a deeper buried cavity. The table summarizes quantitative descriptors of the active sites, including pocket volume and hydrophobicity calculated with Fpocket (Le Guilloux et al. 2009), together with SASA metrics calculated from the predicted structures (https://proteiniq.io/app/sasa‐calculator). These analyses quantitatively support the differences in active‐site accessibility and geometry between both enzymes. The protein structures are displayed at a common scale for visualization of the active sites; therefore, their relative sizes are not proportional.
**Figure S6:** Full multiple sequence alignment (MSA) of consensus disordered proteins generated with FoldMason. Sequences are ordered according to the guide tree, and residues are coloured using the CLUSTAL colour scheme.
**Figure S7:** Guide tree derived from the reduced MSA. Proteins exhibiting catalytic activity form an early‐diverging branch relative to the remaining sequences.
**Figure S8:** Zoomed‐in view of the MSA. The Figure highlights the loop region that forms part of the main substrate‐binding pocket in the consensus proteins. Sequences are ordered according to the guide tree, and residues are coloured using the CLUSTAL colour scheme.
**Figure S9:** PET degradation product profiles generated by PETases from different clusters. (a) Lip_MRD9_ and Lip_Bv_ comparative chromatograms. (b) Lip_Sh1_, Lip_Sh2_ and Lip_Sh3_ comparative chromatograms. (c) Summary table of degradation product profiles of all PET‐hydrolysing enzymes. Sample collected at given times of reaction were dissolved in methanol and were analysed by RP‐HPLC–MS in a Waters Alliance e2695 Separations Module coupled to a Waters 2998 Photodiode Array Detector and coupled to a single quadrupole Waters Acquity QDa mass spectrometer (Waters Cromatografía, SA; Barcelona, Spain). Compounds were analysed by reverse phase chromatography using a 2.1 mm × 5 mm Waters Sunfire C_18_ RP precolumn and 2.1 mm × 100 mm Waters Atlantis T3 C_18_ RP column, operating at 0.35 mL min^−1^. The injection volume of sample was 6 μL. Compounds were eluted using a 10 min gradient from 15% to 95% solvent A‐B (solvent A: acetonitrile, solvent B: water) which includes a 5% fixed percentage of solvent C (solvent C: 2% formic acid in water), being the concentration of formic acid in all the gradient a 0.1%. After the separation, the sample was analysed by optical detection from 190 to 700 nm with a resolution of 1.2 nm (Waters 2998 PDA Detector) and by mass analyser (Waters Acquity QDa Detector) using an Electrospray (ES) Interface in Positive and Negative Mode with a Capillary Voltage of 0.8 kV and a Cone Voltages of 20 V and 40 V, being the Probe Temperature of 600°C and the Source Temperature of 120°C. Compounds were detected in Full Scan MS from 85 to 1250 m/z, with automated mass resolution control of 0.7 Da. All others original data were provided by the Unidad de Análisis Instrumental from the Instituto de Química Médica (IQM‐CSIC), Spain; raw data can be provided upon request by LC–MS Unit. Original MS/MS files in Data S1. The files can be analysed with the software MassLynx v4.1 (Waters) to obtain the mass spectra of each degradation product and their molecular weight.
**Figure S10:** MS/MS analysis of nPET degradation products generated by Lip_MRD9_. Sample was taken at 4 h of reaction at 60°C. Main products eluting in the HPLC analysis ionized at m/z 357 (C_18_H_14_O_8_), at m/z 401 (C_20_H_18_O_9_), at m/z 445 (C_20_H_18_O_9_), at m/z 549 (C_28_H_22_O_12_), at m/z 593 (C_30_H_26_O_13_), at m/z 741 (C_38_H_30_O_16_), at m/z 786 (C_40_H_34_O_17_) and at m/z 978 (C_50_H_42_O_21_). These findings are consistent with these products being TET, TETE, ETETE (a), TETET, TETETE (b), TETETET, TETETETE (c) and TETETETETE (d).
**Figure S11:** MS/MS analysis of nPET degradation products generated by Lip_Bv_. Sample was taken at 4 h of reaction at 60°C. Main products eluting in the HPLC analysis ionized at m/z 357 (C_18_H_14_O_8_), at m/z 401 (C_20_H_18_O_9_), at m/z 440 (unknown), at m/z 445 (C_20_H_18_O_9_), at m/z 549 (C_28_H_22_O_12_), at m/z 593 (C_30_H_26_O_13_), at m/z 741 (C_38_H_30_O_16_), at m/z 786 (C_40_H_34_O_17_) and at m/z 934 (C_48_H_38_O_20_). These findings are consistent with these products being TET, TETE, ETETE (a), TETET, TETETE (b), unknown (c) TETETET, TETETETE (d) and TETETETET (e).
**Figure S12:** MS/MS analysis of nPET degradation products generated by Lip_Sh1_. Sample was taken at 6 h of reaction at 40°C. Main products eluting in the HPLC analysis ionized at m/z 357 (C_18_H_14_O_8_), at m/z 401 (C_20_H_18_O_9_), at m/z 549 (C_28_H_22_O_12_), at m/z 741 (C_38_H_30_O_16_), at m/z 786 (C_40_H_34_O_17_) and at m/z 978 (C_50_H_42_O_21_). These findings are consistent with these products being TET, TETE (a), TETETE (b), TETETET, TETETETE (c) and TETETETETE (d).
**Figure S13:** MS/MS analysis of nPET degradation products generated by Lip_Sh2_. Main product eluting in the HPLC analysis ionized at m/z 357 (C_18_H_14_O_8_), consistent with this product being TET (a).
**Figure S14:** MS/MS analysis of nPET degradation products generated by Lip_Sh3_. Sample was taken at 6 h of reaction at 40°C. Main products eluting in the HPLC analysis ionized at m/z 357 (C_18_H_14_O_8_), at m/z 401 (C_20_H_18_O_9_) and at m/z 445 (C_20_H_18_O_9_). These findings are consistent with these products being TET, TETE and ETETE (a).
**Figure S15:** Phylogenetic relationships among PET‐hydrolysing α/β‐hydrolases. A total of 3752 protein sequences were included in the analysis, comprising experimentally validated PETases, canonical PETases, validated true lipases lacking PET‐hydrolysing activity, predicted PETase homologues identified in this study and the PET‐hydrolysing true lipases characterized here. Sequences were aligned using MAFFT v7.525, and the resulting multiple sequence alignment was trimmed with trimAl v1.4.1 using the ‐gt 0.7 option. A maximum‐likelihood phylogenetic tree was reconstructed with IQ‐TREE v3.1.3 using 1000 bootstrap replicates. Nodes with bootstrap support values ≥ 80 are indicated by black circles, whereas nodes with support values < 80 are indicated by white circles. Tip colours indicate the enzyme class as shown in the legend, and enlarged tip symbols identify representative enzymes discussed in the text. The tree shows that the PET‐hydrolysing true lipases identified in this study form lineages distinct from canonical PETases, supporting the independent emergence of PET‐hydrolysing activity in multiple α/β‐hydrolase families.
**Data S1:** Raw mass spectrometry files supporting the identification of PET‐derived intermediates. The data in the raw folder can be analysed with the software MassLynx V4.1 (Waters) to obtain the mass spectra of PET degradation oligomers. The full dataset is stored and accessible through the following Zenodo link: https://doi.org/10.5281/zenodo.20524881.
**Data S2:** Raw HPLC chromatograms used for the quantification of PET hydrolysis products. Files are provided in .txt format corresponding to the data extracted with Varian ProStar software (Varian Inc., Palo Alto, California, USA). The full dataset is stored and accessible through the following Zenodo link: https://doi.org/10.5281/zenodo.20524881.
**Data S3:** Sequence similarity network file reconstructed with Cytoscape. The network file is provided in .net format and can be directly imported into Cytoscape (version 3.0 or later recommended) for visualization and further network analysis. The file is stored and accessible through the following Zenodo link: https://doi.org/10.5281/zenodo.20524881.
**Data S4:** Raw and processed datasets used for lipid substrate hydrolysis, PET hydrolysis and determination of enzyme pH and temperature optima. (a) Lipid substrate hydrolysis rate calculations. Datasets include the absorbance at 550 nm using NEFA kit and the corresponding specific activity values after incubation of different triglycerides, oils and esters with all of the purified enzymes. Data are provided in triplicates. Related to Table 3 and Table 4. (b) PET hydrolysis rate calculations. Datasets include concentrations of degradation products and the corresponding specific activity values obtained after incubation of nPET and pPET with purified enzymes at 40°C and 60°C. Quantification was performed by HPLC using calibration curves (original chromatograms in Data S2). Data are provided in triplicates, with both raw and processed values (including applied dilutions or concentration factors). Related to Table 4. (c) Determination of enzyme pH and temperature optima of Lip_Sh1_ and Lip_Bv_. Datasets include the absorbance at 550 nm using Tri‐C8 and NEFA kit (temperature) or at 405 nm using *p*NPB (pH) as substrates and the corresponding relative activity values after incubation. Data are provided in triplicates. Related to Figure 3. Raw and processed datasets are provided in spreadsheet format, including replicate measurements and calculated values used for data analysis. These files enable full reproduction of the enzymatic activity calculations and figures reported in the manuscript. The full dataset is stored and accessible through the following Zenodo link: https://doi.org/10.5281/zenodo.20524880.

## Data Availability

The PELE simulations for the lipid and PET ligands have been deposited in a public repository under the identifier https://doi.org/10.5281/zenodo.19846122. To use the archive, download the file and extract its contents to a local directory using appropriate software. The directory contains separate folders for each simulation, together with the corresponding input, output and README files describing the simulation setup and analysis. Due to the size and complexity of the datasets, Tables S1 and S2 have been deposited in Zenodo under the identifier https://doi.org/10.5281/zenodo.20524880. The Zenodo repository also contains the supplementary datasets and documentation required to reproduce the analyses reported in this study. Raw mass spectrometry files supporting the identification of PET‐derived intermediates are available in Data S1. Raw HPLC chromatograms used for product quantification are provided in Data S2. The complete Cytoscape‐compatible network file used for sequence similarity network reconstruction and visualization is provided in DataS3. Processed and unprocessed datasets used for lipid substrate hydrolysis rate calculations are available in Data S4a. Processed and unprocessed datasets used for PET hydrolysis rate calculations are available in Data S4b. Raw absorbance measurements and processed datasets underlying the determination of enzyme pH and temperature optima are available in Data S4c. The Zenodo repository contains separate folders for MS and HPLC files, Tables S1 and S2, network files, raw experimental datasets and accompanying README documentation. These datasets enable full reproduction of the computational analyses, enzymatic activity calculations, PET degradation product quantification and figures reported in this study. Any additional information required to reanalyse the data reported in this paper is available from the corresponding authors upon reasonable request.
