# Supplementary material for: Structure‐Guided Extremophile Genome Mining Expands the PETase Landscape and Reveals PET‐Hydrolysing True Lipase Lineages
Source: Microb Biotechnol. 2026 Aug 21;19(8):e70432. doi: 10.1111/1751-7915.70432 (PMC13498906; doi:10.1111/1751-7915.70432)
Supplement: Supplementary file 1 [file MBT2-19-e70432-s001.docx]

# Structure-guided extremophile genome mining expands the PETase landscape and reveals PET-hydrolysing true lipase lineages

Rubén Muñoz-Tafalla^1,2^ (0000-0003-4380-4888), José M. González-Romero^1,3^ (0009-0004-6552-0709), Paula Vidal^3^ (0000-0001-9180-0895), Laura Fernandez-Lopez^3^ (0000-0001-8861-3191), Ana Robles-Martín^2^ (0000-0002-6377-6338), David Almendral^3^ (0000-0002-1466-1067), José L. Gonzalez-Alfonso^3^ (0000-0002-3396-7985), Francisco J. Plou^3^ (0000-0003-0831-893X), Manuel Ferrer^3^ (0000-0003-4962-4714), Víctor Guallar^2,4,*^ (0000-0002-4580-1114), Rafael Bargiela^3,*^ (0000-0003-1442-3269)

^1^PhD program in Biotecnology, Faculty of Pharmacy and Food Sciences, Universitat de Barcelona (UB), 08028 Barcelona, Spain | ^2^Barcelona Supercomputing Center (BSC), 08010 Barcelona, Spain | ^3^Instituto de Catalisis y Petroleoquimica (ICP), CSIC, 28049 Madrid, Spain | ^4^Institució Catalana de Recerca i Estudis Avançats (ICREA), 08010 Barcelona, Spain

## Supplementary material

| **Supplementary Tables** | 3-6 |
| --- | --- |
| **Table S1** | 3 |
| **Table S2** | 4 |
| **Table S3** | 5-6 |

| **Supplementary Figures** | 7-22 |
| --- | --- |
| **Figure S1** | 7 |
| **Figure S2** | 8-9 |
| **Figure S3** | 10 |
| **Figure S4** | 11 |
| **Figure S5** | 12 |
| **Figure S6** | 13 |
| **Figure S7** | 14 |
| **Figure S8** | 15 |
| **Figure S9** | 16 |
| **Figure S10** | 17 |
| **Figure S11** | 18 |
| **Figure S12** | 19 |
| **Figure S13** | 20 |
| **Figure S14** | 21 |
| **Figure S15** | 22 |

| **Supplementary Data** | 23-26 |
| --- | --- |
| **Data S1** | 23 |
| **Data S2** | 24 |
| **Data S3** | 25 |
| **Data S4** | 26 |

***Table S1****.* ***Reference and candidate true lipases identified during the genome mining workflow.*** *The table summarizes the datasets used for the identification of candidate true lipases from extremophilic genomes. Reference lipase sequences were compiled and employed as query sequences for similarity searches against the GTDB and additional protein databases. a) Reference dataset of true lipases compiled to be used as reference queries for BLAST searches. b) List of genomes retrieved from the GTDB selected possible extremophiles to search lipases. c) Initial list of selected sequences from extremophiles with at least 60% of identity percentage and minimum alignment length of 100 residues with the reference lipases. d) Filtered list of 50 presumptive true lipases identified, after signal peptide detection and reducing sequence redundancy, further preselected candidates for structural and functional analysis. Metadata and sequence features of candidate enzymes identified in this study, including GTDB genome IDs, UniProt matches, sequence identity, signal peptide prediction, sequences used for gene synthesis, environmental origin, taxonomic classification, catalytic triad residues, and predicted structural features. e) Final selected candidates after structural and functional analysis. Sequence features of candidate enzymes identified in this study, including GTDB genome IDs, UniProt matches, sequence identity (%) and alignment length. It shows original and synthetic protein sequences (signal peptides in blue, SignalP-5.0), pET-45(+) plasmid constructs synthesized by GenScript Biotech (EG Rijswijk, The Netherlands; ref. U063CUMTG0, U790YVJEG0 and U5150FMPG0), antibiotic resistance (ampicillin), inducer (IPTG), molecular weights (calculated with ExPASy;* [*https://web.expasy.org/compute_pi/*](https://web.expasy.org/compute_pi/)*), catalytic triad residues, predicted structural features, environmental origin and extremophily and taxonomic classification. Due to the size and complexity of the dataset, Table S1 is provided as a separate Microsoft Excel (.xlsx) file, stored and accessible through the following Zenodo link: https://doi.org/10.5281/zenodo.20524880. All worksheets are labelled according to the workflow stages described in the manuscript and can be accessed using standard spreadsheet software.*

***Table S2.*** ***Reference PETases and candidate PETase homologuesequences identified from extremophilic genomes.*** *This table summarizes the datasets used for the identification of putative PETases from extremophilic microbial genomes. Experimentally characterized PETase sequences were compiled and employed as query sequences for homology-based searches against the GTDB and other protein databases. a) Reference dataset of experimentally validated PETases used as queries for similarity searches. b) Set of 1,322 candidate PETase homologue sequences identified from 18,082 extremophilic microbial genomes retrieved from the GTDB. Metadata and sequence features are provided for each candidate, including GTDB genome ID, UniProt matches, sequence identity, taxonomic classification, and environmental origin. c) List of sequences for SSN. Owing to the large size of the dataset, Table S2 is provided as a separate Microsoft Excel (.xlsx) file, stored and accessible through the following Zenodo link: https://doi.org/10.5281/zenodo.20524880. The file contains multiple worksheets corresponding to the different stages of the PETase mining workflow and can be viewed using standard spreadsheet software.*

**Table S3.** Clustal Omega pairwise distances of proteins belonging to the Lip_Bv_ and Lip_Sh1_ SSN clusters relative to their reference sequences.

| **Sequence ID** | **Clustal Omega Distance to Lip_Bv_** |
| --- | --- |
| MDP4565604.1 | 0.25 |
| OJH64723.1 | 0.066038 |
| OUL02930.1 | 0.051887 |
| PJZ01618.1 | 0 |
| PRR93548.1 | 0.254717 |
| PRS01100.1 | 0.254717 |
| PSA89690.1 | 0.216981 |
| PSA91355.1 | 0.212264 |
| QBG54668.1 | 0.235849 |
| QEK64405.1 | 0.245283 |
| QKS03084.1 | 0.113208 |
| UDB48739.1 | 0.245283 |
| UDB53012.1 | 0.254717 |
| UHH07698.1 | 0.066038 |
| UPV79481.1 | 0.04717 |
| ARC64934.1 | 0.338235 |
| GCA_948330225.1_WGS_ASS_CCMM_B951_genomic_03397 | 0.355769 |
| GCA_948330315.1_WGS_ASS_CCMM_B774_genomic_00908 | 0.350962 |
| GCA_948330325.1_WGS_ASS_CCMM_B969_genomic_03136 | 0.352941 |
| GCA_948330375.1_WGS_ASS_CCMM_B943_genomic_00297 | 0.343137 |
| MBL7474587.1 | 0.355769 |
| MBS2761464.1 | 0.338235 |
| QII49680.1 | 0.357843 |
| UZN54974.1 | 0.352941 |
| WFA03588.1 | 0.28436 |
| AIU80440.1 | 0.245283 |
| ERH50102.1 | 0.245283 |
| MCY0091533.1 | 0.245283 |
| OPD40343.1 | 0.240566 |
| PQB10077.1 | 0.245283 |
| QEK96742.1 | 0.245283 |
| QRD35341.1 | 0.245283 |
| UOM42820.1 | 0.245283 |
| GCA_002320995.1_ASM232099v1_genomic_00014 | 0.462264 |
| URM44798.1 | 0.240566 |
| AIK49165.1 | 0.221698 |
| AKE22080.1 | 0.056604 |
| APH67194.1 | 0.051887 |
| GCA_000444215.1_BacSafVK_genomic_02672 | 0.25 |
| GCA_025460705.1_ASM2546070v1_genomic_03669 | 0.075472 |
| Lip_MRD9_ | 0.25 |
| MBR0618917.1 | 0.254717 |
| MCG8397989.1 | 0.221698 |
| MCV0025519.1 | 0.113208 |
| MDK4256634.1 | 0.231132 |
| MDP4525660.1 | 0.108491 |
| **Sequence ID** | **Clustal Omega Distance to Lip_Sh1_** |
| Lip_Sh2_ | 0.180132 |
| Lip_Sh3_ | 0.178808 |

**Figure S1.** **Best docking poses of candidate Lip_Se_, which showed the highest CBFE for both substrates**. a) Docked PET tetramer and b) docked Tri-C10. In both panels, the protein is shown as a yellow cartoon, highlighting the catalytic triad residues and the substrate represented in stick format. Hydrogen-bond interactions among the catalytic triad residues are shown in pink, while the distance between the catalytic serine and the closest ester carbon of the substrate is indicated in cyan. These representations illustrate the catalytic poses selected for the PELE simulations, showing the reactive substrate atoms positioned near the catalytic serine and providing a visual example of a productive binding mode for each substrate.

**Figure S2.** **Energy interactions of candidate enzymes**. a) Interaction energy profiles for the 23 candidate enzymes using Tri-C10 as the substrate. Each scatter plot shows all poses sampled during the simulation for a given protein. The x-axis represents the distance between the reactive oxygen atom of the catalytic serine and the closest ester bond of the triglyceride ligand (Å), while the y-axis shows the interaction energy (kcal mol⁻¹). The color scale indicates the total energy of each pose (kcal mol⁻¹). b) Ranking based on the calculated CBFE. The black arrow indicates the selected candidates for the next stage.

**Figure S3.** **PELE simulation results obtained using the PET tetramer as the ligand.** Scatter plots show all poses sampled throughout the simulations for each protein. The x-axis indicates the distance between the reactive oxygen atom of the catalytic serine and the closest non-terminal ester bond of the polymeric ligand (Å), while the y-axis shows the interaction energy (kcal mol⁻¹). The color scale indicates the total energy of each pose (kcal mol⁻¹). a) Interaction energy profiles for the three benchmark PETases: Fast-PETase, HiC, and LCC-ICCG. b) Interaction energy profiles for the 15 selected candidate enzymes. c) Ranking based on the calculated Catalytic Binding Free Energy (CBFE). The black arrow indicates the highest CBFE value among the benchmark PETases; therefore, only candidates with lower CBFE values were selected for experimental validation.

**Figure S4.** **SDS–PAGE analysis of recombinant enzymes.** 12% polyacrylamide SDS PAGE of enzymes. Lane 1: total proteins. Lane 2: soluble protein. Lane 3: pure proteins 10 µg.

***Figure S5.*** ***Structural comparison of the active sites of Lip_Bv_ and Lip_Sh1_.***  *Surface representations of the Alphafold predicted structures highlighting the active-site residues (colored). Front and side/cross-sectional views illustrate the distinct active-site architectures of the two enzymes, with Lip_Bv_ displaying an open surface groove and Lip_Sh1_ a deeper buried cavity. The table summarizes quantitative descriptors of the active sites, including pocket volume and hydrophobicity calculated with Fpocket (Le Guilloux et al., 2009), together with SASA metrics calculated from the predicted structures (https://proteiniq.io/app/sasa-calculator). These analyses quantitatively support the differences in active-site accessibility and geometry between both enzymes. The protein structures are displayed at a common scale for visualization of the active sites; therefore, their relative sizes are not proportional.*

**Figure 6.** **Full multiple sequence alignment (MSA) of consensus disordered proteins generated with FoldMason**. Sequences are ordered according to the guide tree, and residues are colored using the CLUSTAL color scheme.

**Figure S7.** **Guide tree derived from the reduced MSA.** Proteins exhibiting catalytic activity form an early-diverging branch relative to the remaining sequences.

**Figure S8.** **Zoomed-in view of the MSA.** The Figure highlights the loop region that forms part of the main substrate-binding pocket in the consensus proteins. Sequences are ordered according to the guide tree, and residues are colored using the CLUSTAL color scheme.

**Figure S9.** **PET degradation product profiles generated by PETases from different clusters.** a) Lip_MRD9_ and Lip_Bv_ comparative chromatograms. b) Lip_Sh1_, Lip_Sh2_ and Lip_Sh3_ comparative chromatograms. c) Summary table of all PETases degradation products’ degradation profiles. Sample at given times of reaction were solved in methanol and were analyzed by RP-HPLC-MS in a Waters Alliance e2695 Separations Module coupled to a Waters 2998 Photodiode Array Detector and coupled to a single quadrupole Waters Acquity QDa mass spectrometer (Waters Cromatografía, SA; Barcelona, Spain). Compounds were analyzed by reverse phase chromatography using a 2.1 mm × 5 mm Waters Sunfire C_18_ RP precolumn and 2.1 mm × 100 mm Waters Atlantis T3 C_18_ RP column, operating at 0.35 mL min^-1^. The injection volume of sample was 6 µL. Compounds were eluted using a 10 min gradient from 15 to 95% solvent A-B (solvent A: acetonitrile, solvent B: water) which includes a 5% fixed percentage of solvent C (solvent C: 2% formic acid in water), being the concentration of formic acid in all the gradient a 0.1%. After the separation, the sample was analyzed by optical detection from 190-700nm with a resolution of 1.2nm Waters 2998 PDA Detector) and by mass analyzer (Waters Acquity QDa Detector) using an Electrospray (ES) Interface in Positive and Negative Mode with a Capillary Voltage of 0.8kV and a Cone Voltages of 20V y 40V, being the Probe Temperature of 600 °C and the Source Temperature of 120 °C. Compounds were detected in Full Scan MS from 85 to 1250 m/z, with automated mass resolution control of 0.7Da. All others original data provided by the Unidad de Análisis Instrumental from the Instituto de Química Médica (IQM-CSIC), Spain; raw data can be provided upon request by LC-MS Unit. Original MS/MS files in Data S1. The files can be analyzed with the software MassLynx v4.1 (Waters) to obtain the mass spectra of each degradation product and their molecular weight.

**Figure S10.** **MS/MS analysis of nPET degradation products generated by Lip_MRD9_.** Sample was taken at 4 hours of reaction at 60 °C. Main products eluting in the HPLC analysis ionized at m/z 357 (C_18_H_14_O_8_), at m/z 401 (C_20_H_18_O_9_), at m/z 445 (C_20_H_18_O_9_), at m/z 549 (C_28_H_22_O_12_), at m/z 593 (C_30_H_26_O_13_), at m/z 741 (C_38_H_30_O_16_), at m/z 786 (C_40_H_34_O_17_), and at m/z 978 (C_50_H_42_O_21_). These findings are consistent with these products being TET, TETE, ETETE (a), TETET, TETETE (b), TETETET, TETETETE (c), and TETETETETE (d).

**Figure S11.** **MS/MS analysis of nPET degradation products generated by** **Lip_Bv_.** Sample was taken at 4 hours of reaction at 60 °C. Main products eluting in the HPLC analysis ionized at m/z 357 (C_18_H_14_O_8_), at m/z 401 (C_20_H_18_O_9_), at m/z 440 (unknown), at m/z 445 (C_20_H_18_O_9_), at m/z 549 (C_28_H_22_O_12_), at m/z 593 (C_30_H_26_O_13_), at m/z 741 (C_38_H_30_O_16_), at m/z 786 (C_40_H_34_O_17_), and at m/z 934 (C_48_H_38_O_20_). These findings are consistent with these products being TET, TETE, ETETE (a), TETET, TETETE (b), unknown (c) TETETET, TETETETE (d), and TETETETET (e).

**Figure S12.** **MS/MS analysis of nPET degradation products generated by Lip_Sh1_.** Sample was taken at 6 hours of reaction at 40 °C. Main products eluting in the HPLC analysis ionized at m/z 357 (C_18_H_14_O_8_), at m/z 401 (C_20_H_18_O_9_), at m/z 549 (C_28_H_22_O_12_), at m/z 741 (C_38_H_30_O_16_), at m/z 786 (C_40_H_34_O_17_), and at m/z 978 (C_50_H_42_O_21_). These findings are consistent with these products being TET, TETE (a), TETETE (b), TETETET, TETETETE (c), and TETETETETE (d).

**Figure S13.** **MS/MS analysis of nPET degradation products generated by Lip_Sh2_.** Main product eluting in the HPLC analysis ionized at m/z 357 (C_18_H_14_O_8_), consistent with this product being TET (a).

**Figure S14.** **MS/MS analysis of nPET degradation products generated by Lip_Sh3_.** Sample was taken at 6 hours of reaction at 40 °C. Main products eluting in the HPLC analysis ionized at m/z 357 (C_18_H_14_O_8_), at m/z 401 (C_20_H_18_O_9_), and at m/z 445 (C_20_H_18_O_9_). These findings are consistent with these products being TET, TETE and ETETE (a).

**Figure S15.** **Phylogenetic relationships among PET-hydrolysing α/β-hydrolases.** A total of 3,752 protein sequences were included in the analysis, comprising experimentally validated PETases, canonical PETases, validated true lipases lacking PET-hydrolysing activity, predicted PETase homologues identified in this study, and the PET-hydrolysing true lipases characterized here. Sequences were aligned using MAFFT v7.525, and the resulting multiple sequence alignment was trimmed with trimAl v1.4.1 using the -gt 0.7 option. A maximum-likelihood phylogenetic tree was reconstructed with IQ-TREE v3.1.3 using 1,000 bootstrap replicates. Nodes with bootstrap support values ≥80 are indicated by black circles, whereas nodes with support values <80 are indicated by white circles. Tip colours indicate the enzyme class as shown in the legend, and enlarged tip symbols identify representative enzymes discussed in the text. The tree shows that the PET-hydrolysing true lipases identified in this study form lineages distinct from canonical PETases, supporting the independent emergence of PET-hydrolysing activity in multiple α/β-hydrolase families.

***Data S1.*** ***Raw mass spectrometry files supporting the identification of PET-derived intermediates.*** *The data in the raw folder can be analyzed with the software MassLynx V4.1 (Waters) to obtain the mass spectra of PET degradation oligomers. The mass spectrometry files can be analyzed with the software MassLynx V4.1. The full dataset is stored and accessible through the following Zenodo link: https://doi.org/10.5281/zenodo.20524881.*

***Data S2.*** ***Raw HPLC chromatograms used for the quantification of PET hydrolysis products.*** *Files are provided in .txt format corresponding to the data extracted with Varian ProStar software (Varian Inc., Palo Alto, California, USA). The full dataset is stored and accessible through the following Zenodo link: https://doi.org/10.5281/zenodo.20524881.*

***Data S3. Sequence similarity network file reconstructed with Cytoscape.*** *The network file is provided in .net format and can be directly imported into Cytoscape (version 3.0 or later recommended) for visualization and further network analysis. The file is stored and accessible through the following Zenodo link: https://doi.org/10.5281/zenodo.20524881.*

***Data S4. Raw and processed datasets used for lipid substrate hydrolysis, PET hydrolysis, and determination of enzyme pH and temperature optima.*** *a) Lipid substrate hydrolysis rate calculations. Datasets include the absorbance at 550 nm using NEFA kit and the corresponding specific activity values after incubation of different triglycerides, oils and esters with all of the purified enzymes. Data are provided in triplicates. Related to Table 3 and Table 4. b) PET hydrolysis rate calculations. Datasets include concentrations of degradation products and the corresponding specific activity values obtained after incubation of nPET and pPET with purified enzymes at 40 and 60 °C. Quantification was performed by HPLC using calibration curves (original chromatograms in Data S2). Data are provided in triplicates, with both raw and processed values (including applied dilutions or concentration factors). Related to Table 4. c) Determination of enzyme pH and temperature optima of Lip_Sh1_ and Lip_Bv_. Datasets include the absorbance at 550 nm using Tri-C8 and NEFA kit (temperature) or at 405 nm using pNPB (pH) as substrates and the corresponding relative activity values after incubation. Data are provided in triplicates. Related to Figure 3. Raw and processed datasets are provided in spreadsheet format, including replicate measurements and calculated values used for data analysis. These files enable full reproduction of the enzymatic activity calculations and figures reported in the manuscript. The full dataset is stored and accessible through the following Zenodo link: https://doi.org/10.5281/zenodo.20524881.*
